# The Effect of Iranian Propolis on Glucose Metabolism, Lipid Profile, Insulin Resistance, Renal Function and Inflammatory Biomarkers in Patients with Type 2 Diabetes Mellitus: A Randomized Double-Blind Clinical Trial

**DOI:** 10.1038/s41598-019-43838-8

**Published:** 2019-05-13

**Authors:** Mehrnoosh Zakerkish, Maryam Jenabi, Narjes Zaeemzadeh, Ali Asghar Hemmati, Niloofar Neisi

**Affiliations:** 1 Diabetes research center, Health research institute, Ahvaz Jundishapur University of Medical Sciences, Ahvaz, Iran; 2 Department of Pharmacology, School of Pharmacy, Ahvaz Jundishapur University of Medical Sciences, Ahvaz, Iran; 3 Medicinal Plant Research Center, School of pharmacy, Ahvaz Jundishapur University of Medical Sciences, Ahvaz, Iran; 4 Department of Virology, School of Medicine, Ahvaz Jundishapur University of Medical Sciences, Ahvaz, Iran

**Keywords:** Type 2 diabetes, Randomized controlled trials

## Abstract

Propolis is a natural product with many biological properties including hypoglycemic activity and modulating lipid profile. The present study was designed to evaluate the effect of Iranian propolis extract on glucose metabolism, Lipid profile, Insulin resistance, renal and liver function as well as inflammatory biomarkers in patients with type 2 diabetes mellitus (T2DM). A double-blind, placebo-controlled clinical trial was conducted. The duration of the study lasted 90 days. Patients with T2DM were recruited and randomly divided into an Iranian propolis group (1000 mg/day) (n = 50) and a placebo group (n = 44). There was a significant decrease in the serum levels of glycosylated hemoglobin (HbA1c), 2-hour post prandial (2hpp), insulin, homeostasis model assessment-insulin resistance (HOMA-IR), homeostasis model assessment of β-cell function (HOMA-β), High sensitive C-reactive protein (hs-CRP), tumor necrosis factor-α (TNF-α). However, there was a notable elevation in the serum HDL-C in the propolis group compared with the placebo group. In addition, a notable reduction in serum liver transaminase (ALT and AST) and blood urea nitrogen (BUN) concentrations in the propolis group was observed. Iranian propolis has beneficial effects on reducing post prandial blood glucose, serum insulin, insulin resistance, and inflammatory cytokines. It is also a useful treatment for preventing the liver and renal dysfunction, as well as, elevating HDL-C concentrations in patients with T2DM.

## Introduction

Diabetes Mellitus (DM) is a prevalent chronic disorder characterized by an elevated blood glucose concentration. It has two types (1 and 2) which are summarized as T1DM and T2DM, respectively. T1D is an autoimmune disease, with absolute insulin deficiency and starting in youth. While T2D is preceded by insulin resistance, hyperinsulinemia and beta-cell exhaustion^1,2^

The prevalence of diagnosed diabetes is dramatically increasing worldwide. The International Diabetes Federation reported that the number of diabetic patients, in 2011was 366 million and they predict that by 2030 that number will be 439 million. This is due to an increase in sedentary lifestyle and the consequent prevalence of obesity^3^.

There is a higher proportion of type 2 diabetes and poorly controlled type 2 diabetes is associated with microvascular (retinopathy and nephropathy), macrovascular, and non-vascular (neuropathy) complications. This leads to poor prognosis and a significant decrease in life expectancy^4^. It is reported recently that, the mortality rate of diabetes is 2 to 4 times higher than that of cardiovascular disease^5^. In addition to the elevated rates of morbidity and mortality associated with T2DM, economically the total cost of care and management is very high^6^.

Previous studies revealed that diabetes increases the production of inflammatory mediators. These include, tumor necrosis factor-α (TNF-α), interleukin-6 (IL-6) and interleukin 1 (IL-1) which are the main cytokines involved in the pathogenesis of the disease via direct cytotoxic effect, induction of free radicals and apoptosis activating pathway^7–12^.

Additionally, persistent hyperglycemia causes oxidative stress which in turn, deteriorates β-cells function and decreases the sensitivity of peripheral tissues in response to insulin^13–16^.

Researchers are now interested in the health benefits of alternative medicinal foods with natural antioxidant bioactive compounds as new adjunctive pharmaceuticals for reducing T2DM complications^17^. Natural bioactive products may be able to control blood glucose concentration and decrease the risk of complications^18^.

Propolis is a natural resinous hive product that honeybees (*Apis mellifera L*.) collect from various plant sources. They mix it with both their salivary gland enzymes and wax, and then use it to seal holes in their honeycombs, smoothing out internal walls and protecting the entrance against intruders^19^. It has been used in traditional medicine, all over the world, and dates back to at least 300 BC, due to its distinctive biological and antioxidant properties^20–22^.

Propolis has a very complex chemical composition due to several phytogeographic characteristics that are inherent to the varying plant habitats and plant types that bees choose from to produce propolis (14, 19). These characteristics include: vegetation, season, and the environmental conditions of the collection site. This aspect makes universal standardization of propolis nearly impossible^23^. Therefore, it is important to mention the country of origin that the propolis was derived from. Over 300 active constituents have been found in propolis. In general they are mostly a mixture of phenols (e.g., flavonoid, polyphenol, and aromatic compounds), terpenes, amino acids, vitamins, sugars, and elements^24^. The most recent study has reported that Iranian propolis is rich in flavonoids, phenolic and terpene compounds that are similar to Cuban, Brazilian and Egyptian propolis^25^. A chemical analysis of the Iranian propolis performed during the present study, determined that the major components were flavonoids and phenolic acid esters. It should be mentioned that despite the differences in chemical composition between samples of various geographical locations, they usually show comparable pharmacological properties^19^.

Many studies have established the vast biological and pharmacolocial properties of propolis. The following medicinal properties have been found: antimicrobial^26–28^, immunomudulatory^29^, antitumor^30–32^, anti-inflammatory^33–35^, antioxidant^36–38^, antiviral^39,40^, antifungal^41,42^, and having hepatoprotective, nephroprotective and pancreatoprotective activities^43–47^. Furtherore, recent studies have revealed that propolis affects hypoglycemic activity, which may positively impact diabetic complications. It also modulates the metabolism of blood lipid levels which leads to a decrease in lipid peroxidation and scavenges the free radicals^6,48–51^. The broad spectrum of biological activity, safety, and longstanding usage makes propolis a potentially useful clinical drug^17,21^.

To the best of our knowledge, the clinical trials concerning propolis efficacy on T2DM are few, with no uniform criteria, and their results are significantly controversial^52–54^. Therefore, further clinical trials are required to clarify the efficacy of propolis on T2DM patients.

The purpose of this study was to evaluate the effect of Iranian propolis extract on glucose metabolism, lipid profile, insulin resistance, renal and liver function as well as inflammatory biomarkers in patients with T2DM.

## Materials and Methods

### Study subjects

#### Enrolled participants

T2DM were screened and enrolled from the Department of Endocrinology, Golestan hospital, Ahvaz, Iran. Patients were between 35–85 years old, receiving treatment with oral hypoglycemic agents. Patients with T2DM were selected based on the criteria classified in the American Diabetes Association^2^: including: A1C ≥ 6.5%, OR; FPG ≥ 126 mg/dl (7.0 mmol/l), OR; 2-h plasma glucose ≥200 mg/dl (11.1 mmol/l), OR; In a patient with classic symptoms of hyperglycemia or hyperglycemic crisis, a random plasma glucose ≥200 mg/dl (11.1 mmol/l).

#### Excluded participants

The patients treated with insulin; severe renal dysfunction [estimated glomerular filtration rate (eGFR) < 30 ml/min/1.73 m^2^]; severe hepatic dysfunction (aspartate aminotransferase (AST) > 100 U/I) or alanine aminotransferase (ALT) > 100 U/I); serious cardiovascular and hematological disease; diabetes diagnosed history >10 years; patients with any type of allergies, and women who are pregnant or lactating were excluded from the study.

### Informed consent

Finally, 100 patients were entered the study. The candidates were informed of the study goals and procedures using a leaflet and a signed written informed consent was obtained. Supplements were licensed by the Iranian Ministry of Health and Medical Education and all procedures were in compliance with the relevant guidelines and regulations. The Ethics Committee of Ahvaz Jundishapur University of Medical Sciences, Ahvaz, Iran, approved the protocol of this study, under the case number, IR.AJUMS.REC.1396.430. The study protocols are available on the Iranian Ministry of Health website (www.irct.ir) under the code: IRCT2016092730008N1, 7/9/2017. The authors confirm that no changes were made to the initial protocols.

### Study design

Iranian propolis capsules used in this study were provided by Shahdine Golha Co. (Isfahan, Iran) and were collected from bee hives located in different parts of the Eastern Azarbayejan province during the fall season and verified by an agricultural organization. Each capsule contained 500 mg Iranian propolis.

Iranian propolis is a poplar type propolis. The chemical profile was characterized by the three parameters: total flavones/flavonols, flavanones/dihydroflavonols, and phenolic compounds which are used as a measure for the amount of active principles^23^. The spectrophotometric assay based on the formation of aluminium chloride complex was applied for quantification of total flavones/flavonols^55^. To measure the amount of flavanones/ dihydroflavonols, a colorimetric method with DNP (2, 4 dinitrophenylhydrazine) was used. Lastly, the total amount of phenolic compounds content was measured using the Folin–Ciocalteu procedure^55^. According to investigation for “typical poplar sample” these amounts should be within the following range: flavones/flavonols: 8 ± 4%, flavanones/dihydroflavonols: 6 ± 2%, total phenolic compounds: 28 ± 9%^23^. Our samples were within these ranges (total flavones and flavonols: 8.4%, total flavanones and dihydroflavonols: 4.6% and total phenolic compounds: 28%).

The present study was a randomized double-blind study. The “Random Allocation” Software was used to allocate patients into either the propolis group or the placebo group. The placebo served as a baseline reference. The details of each patient’s demographics, medical history, and medicines they are currently taking were recorded. Body weight and height, hip and waist circumferences were also measured prior to the study and again at the end. Body mass index was calculated as body weight in kilograms divided by height in meters squared.

The propolis group received Iranian propolis capsules (500 mg twice daily, 30 kcal/day), whereas, the placebo group were given capsules with the same shape, color, and container. The placebo group also followed the same protocol as propolis group (twice daily, 30 kcal/day). The placebo capsules were produced by the same company that manufactured the propolis capsules, included all the ingredients except the active ingredient of propolis. In each group the oral medication was administrated every 12 hours before meals with a glass of water for a period of 90 days. Patients were asked to continue their pervious diet and exercise regimen without any change during the intervention period and this issue reminded to them every week. Fasting and 2-hour post prandial (2hpp) blood samples were collected, in the morning, both at the beginning and the end of the study, from the antecubital vein. The changes in the biochemical parameters and inflammatory cytokines were evaluated.

### Dietary survey

In the final week of the study, experienced interviewers conducted a 3-day dietary survey using a 24 h recall method. The purpose was to compare the dietary intake of energy and nutrients between the two groups. The data were calculated based on Iranian Food Composition which was compiled by the Department of Nutrition, Tehran University of Medical Sciences, Tehran, Iran. Patients were asked not to change the normal diet, activity, and lifestyle during the study.

### Measurement of biochemical parameters

The biochemistry analysis was carried out before and after the intervention at the Diabetes Research Center, Ahvaz Jundishapur University of Medical Sciences, Ahvaz, Iran. Concentration of serum glucose, creatinine (Cr), blood urea nitrogen (BUN), triglyceride (TG), Total cholesterol, high density lipoprotein-Cholesterol (HDL-C), low density lipoprotein-Cholesterol (LDL-C), very low-density lipoprotein-Cholesterol (VLDL-C) and uric acid were determined using a commercial kit (Parsazmoon, Tehran, Iran) and Hitachi multianalyser (Hitachinaka, Japan). Both were used in accordance with the manufacturer’s instructions. The glycosylated hemoglobin (HbA1c) was determined using the Nycocard commercial HbA1c kit (oslo, Norway). Fasting insulin concentration was obtained with the use of enzyme-linked immunosorbent assay (Elisa) commercial kit (Monobind, California, USA). Fasting blood sugar (FBS) and insulin levels were used to calculate homeostasis model assessment-insulin resistance (HOMA-IR) and homeostasis model assessment of β-cell function (HOMA-β) using the following equations: HOMA-IR = glucose (mg/dl)*fasting insulin (µU/ml)/405 & HOMA-β = 20* fasting insulin(µU/ml)/(Fasting glucose(mg/dl)−3.5).

eGFR was calculated using the Cockcroft-Gault Equation: eGFR = ((140-Age)/(serum creatinine)) * (weight/72) for males and for females the eGFR was multiplied by a correction factor of 0.85. Levels of serum IL-1β and TNF-α were established using commercial Elisa kits (Diaclone, Besancon, France). High sensitive C-reactive protein (hs-CRP) was also measured by a commercial Elisa kit (ZellBio, Ulm, Germany).

### Statistical analysis

To calculate the sample size we used the following formula:

$$n=\frac{{({Z}_{(1-\beta )}+{Z}_{(1-\beta /2)})}^{2}({\delta }_{1}^{2}+{\delta }_{2}^{2})}{{({\mu }_{1}-{\mu }_{2})}^{2}}$$

Where, alpha = 0.05, and beta = 0.2.

It showed that 84 diabetic patients would be required for this study. With regard to the attrition rate of 15% the final sample size was considered 100.

According to the results of Fuduka *et al*., the following values were determined: μ1 = 20.7, μ2 = 16.4, δ1 = 3.7, and δ2 = 9.1^54^.

SPSS 16 software was used for all statistical analysis (SPSS Inc., Chicago, IL, USA). The mean values are expressed as mean ± standard deviation and checked for normality using the Kolmogorov-Smirnov test before doing further analysis. As the data were all normal, only parametric tests were used. Baseline information was analyzed using independent sample t-test (Tables 1–3). To evaluate the efficacy of propolis against placebo we used independent sample t-test (Tables 4–8). Paired-sample t-tests were used to compare between baseline and after intervention within groups (Tables 4–8). A result of p < 0.05 was considered statistically significant.

**Table 1:** Demographic and General Characteristic of the Patients at baseline.

Variables	Propolis (n = 50)	Placebo (n = 44)	P-Value
Age, years	55.40 ± 9.09	54.86 ± 8.89	0.77
Gender, male/female	17/33	16/28	0.81
Body height, cm	162.66 ± 8.23	162.82 ± 8.24	0.92
Body Weight, kg	79.16 ± 13.06	77.02 ± 12.48	0.42
BMI, kg/m^2^	30.04 ± 5.41	29.02 ± 4.08	0.31
Duration of diabetes, years	6.36 ± 3.24	6.20 ± 3.18	0.81
Physical activity level	1.53 ± 1.26	1.63 ± 0.76	0.20
Hypertension, −/+	32/18	26/18	0.63
Hyperlipidemia, −/+	19/31	17/27	0.76
Smoking, −/+	43/3	42/2	0.75
Biguanides, −/+	0/50	2/42	0.16
Sulfonylurea, −/+	16/34	20/24	0.19
Pioglitazone, −/+	44/6	40/4	0.65
Alpha-glucosidase inhibitor, −/+	48/2	42/2	0.89
Dipeptiyl peptidase-IV inhibitor, −/+	31/19	33/11	0.18
Meglitinides Derivatives	47/3	42/2	0.757
Systolic blood pressure, mmHg	133.88 ± 14.24	129.98 ± 14.87	0.89
Diastolic blood pressure, mmHg	82.32 ± 7.883	84.27 ± 7.28	0.95

Data are shown as the mean ± standard deviation or number of patients. BMI, body mass index.

**Table 2:** Laboratory Characteristic of Patients at baseline.

Variables	Propolis(n = 50)	Placebo(n = 44)	P-Value
Hemoglobin A1C, %	8.65 ± 1.24	8.78 ± 1.09	0.86
2-hour postprandial, mg/dl	161.6 ± 50.38	168.05 ± 52.61	0.546
Fasting blood glucose, mg/dl	176.6 ± 48.26	169.68 ± 49.42	0.559
Insulin, μIU/ml	14.03 ± 15.43	14.71 ± 12.77	0.82
HOMA-IR	6.03 ± 6.55	6.05 ± 5.02	0.986
HOMAβ	1.76 ± 2	1.91 ± 1.76	0.696
BUN, mg/dl	12.57 ± 3.07	12.84 ± 3.59	0.55
Serum Cr, mg/dl	0.81 ± 0.2	0.84 ± 0.3	0.555
eGFR, ml/min	110.91 ± 43.78	114.31 ± 74.83	0.37
Uric acid, mg/dl	3.82 ± 1.19	3.72 ± 1.54	0.736
Triglycerides, mg/dl	162.8 ± 73.16	164.52 ± 121.46	0.933
Total cholesterol, mg/dl	149 ± 39.62	153 ± 43.91	0.645
HDL-Cholesterol, mg/dl	44.66 ± 8.69	43.98 ± 10.21	0.727
LDL-Cholesterol, mg/dl	71.73 ± 29.48	75.84 ± 26.2	0.485
AST, IU/I	27.42 ± 11.56	25.67 ± 9.98	0.441
ALT, IU/I	29.2 ± 26.26	26.91 ± 19.23	0.635
ALP, IU/I	240.68 ± 119.75	280.55 ± 255.11	0.325
hs-CRP, ng/ml	3589.74 ± 25.20.96	3925.92 ± 2546.47	0.522
TNFα, pg/ml	122.98 ± 115.44	130.24 ± 11.99	0.765
IL-6, pg/ml	32.88 ± 44.61	23.05 ± 22.03	0.37
IL-1β, pg/ml	34.84 ± 32.74	23.72 ± 13.42	0.391

Data are shown as the mean ± standard deviation. HOMA-IR, homeostasis model assessment of insulin resistance; HOMAβ, homeostasis model assessment of β-cell function; BUN, blood urea nitrogen; Cr, creatinine; eGFR, estimated glomerular filtration rate; HDL, high-density lipoprotein; LDL, low-density lipoprotein; AST, aspartate aminotransferase; ALT, alanine aminotransferase; ALP, alkalin phosphatase; TNF-α, tumor necrosis factor-α; hs-CRP, high sensitivity C-reactive protein; IL-1β, interleukin-1β; IL-6, interleukin-6.

**Table 3:** Results of dietary daily survey in patients with T2DM at baseline.

Variables	Propolis (n = 50)	Placebo (n = 44)	P-Value
Energy, kcal	1873.98 ± 129.94	1869.58 ± 120.38	0.87
Protein, g	54.05 ± 6.84	56.19 ± 11.71	0.31
Lipids, g	83.57 ± 12.02	83.22 ± 13.63	0.9
Cholesterol, mg	156.28 ± 77.94	168.90 ± 83.74	0.47
Mono unsaturated fat, g	35.55 ± 5.43	35.43 ± 6.51	0.93
Oleic Fat, g	33.73 ± 5.05	33.81 ± 6.32	0.94
Poly unsaturated fat, g	25.29 ± 5.09	24.98 ± 5.55	0.79
Saturated fat, g	17.32 ± 4.6	17.99 ± 5.75	0.54
DHAomega3, g	0.02 ± 0.06	0.03 ± 0.09	0.46
Epa omega3, g	0.005 ± 0.021	0.009 ± 0.03	0.45
Carbohydrates, g	231.55 ± 34.53	230.31 ± 33.9	0.87
Sugar, g	47.73 ± 22.1	48.76 ± 19.04	0.82
Glucose, g	7.32 ± 4.45	7.03 ± 3.53	0.74
Fructose, g	10.45 ± 6.24	10.06 ± 5.14	0.75
Lactose, g	4.33 ± 3.94	5.43 ± 4.64	0.24
Galactose, g	0.62 ± 0.89	0.7 ± 0.71	0.67
Sucrose, g	20.09 ± 16.89	20.94 ± 11.48	0.79
Maltose, g	1 ± 1.04	0.71 ± 0.81	0.15
Fiber, g	13.29 ± 2.64	14.27 ± 3.73	0.15
Soluble fiber, g	0.27 ± 0.16	0.29 ± 0.19	0.58
Crude Fiber, g	3.25 ± 1.29	3.22 ± 1.07	0.90
Potassium, mg	1744.3 ± 375.37	1826.73 ± 390.36	0.32
Sodium, mg	693.11 ± 271.11	804.22 ± 383.71	0.12
Iron, mg	9.87 ± 1.59	10.44 ± 2.19	0.17
Magnesium, mg	212.51 ± 37.81	224.77 ± 38.95	0.14
Zinc, mg	7.19 ± 1.07	7.41 ± 1.27	0.40
Mangenes, mg	3.26 ± 0.69	3.32 ± 0.69	0.68
Molybdenum, µg	12.1 ± 14.28	16.78 ± 12.31	0.11
Calcium, mg	442.34 ± 167.01	507.14 ± 169.91	0.08
Phosphorus, mg	832.06 ± 127.87	886.33 ± 145.07	0.07
Copper, mg	1.03 ± 0.2	1.04 ± 0.18	0.90
Selenium, mg	0.03 ± 0.02	0.04 ± 0.02	0.04
Chromium, mg	0.02 ± 0.01	0.02 ± 0.01	0.83
Vitamin A, RE	512.26 ± 383.45	491.92 ± 378.75	0.80
Vitamin E, mg	26.64 ± 6.01	26.47 ± 5.67	0.89
Betacaroten, µg	566.11 ± 537.55	694.03 ± 584.77	0.29
Biotin, µg	8.56 ± 4.55	10.2 ± 5.08	0.12
Riboflavin, mg	0.85 ± 0.25	0.98 ± 0.28	0.03
Pyridoxine, mg	1.31 ± 0.39	1.38 ± 0.38	0.41
Thiamin, mg	1.24 ± 0.25	1.31 ± 0.26	0.17
Niacin, mg	16.22 ± 3.01	17.17 ± 4.4	0.26
Folate, µg	257.15 ± 65.91	276.49 ± 64.29	0.17
Pantothenic acid, mg	3.64 ± 0.58	3.92 ± 0.96	0.12
Cobalamin, µg	1.91 ± 0.76	1.99 ± 0.65	0.64
Ascorbic acid, mg	56.48 ± 25.49	68.3 ± 38.25	0.10
Vitamin K, µg	86.5 ± 56.35	93.27 ± 49.23	0.56
Vitamin D, µg	0.37 ± 0.23	0.39 ± 0.27	0.65

Data are shown as the mean ± standard deviation. Whenever the amount of P-Values become less than 0.05 the hypothesis of equal mean between two groups of Propolis and Placebo will be rejected.

**Table 4:** Effect of Iranian propolis on BMI and body weight in T2DM patients after 90 days.

Variables	Propolis(n = 50)		Placebo(n = 44)		P-Value^a^
	Before	After	Before	After	
Weight, kg	79.16 ± 13.06	79.26 ± 12.99	77.02 ± 12.48	77.22 ± 12.48	0.44
BMI, kg/m2	30.04 ± 5.41	30.08 ± 5.38	29.02 ± 4.08	29.09 ± 4.07	0.33

^a^Comparison of change between placebo and Iranian propolis values after 90 days. ^b^P < 0.05 for baseline versus after 90 days within the group. Data are mean ± standard deviation. BMI, body mass index.

**Table 5:** Effect of Iranian propolis on glucose metabolism in T2DM patients after 90 days.

Variables	Propolis(n = 50)		Placebo(n = 44)		P-Value^a^
	Before	After	Before	After	
Hemoglobin A1C, %	8.65 ± 1.24	7.67 ± 1.27^b^	8.78 ± 1.09	8.39 ± 1.15	0.006
2-hour postprandial, mg/dl	161.6 ± 50.38	159.54 ± 49.2	168.05 ± 52.61	223.39 ± 82.04^b^	0.000
Fasting blood glucose, mg/dl	176.6 ± 48.26	171.12 ± 52.86	169.68 ± 49.42	179.34 ± 53.47	0.456
Insulin, μIU/ml	14.03 ± 15.43	7.61 ± 6.69^b^	14.71 ± 12.77	15.46 ± 13.71	0.000
HOMA-IR	6.03 ± 6.55	3.69 ± 2.62^b^	6.05 ± 5.02	6.91 ± 6.7	0.000
HOMAβ	1.76 ± 1.2	1.02 ± 0.69^b^	1.91 ± 1.76	1.85 ± 1.56	0.000

^a^Comparison of change between placebo and Iranian propolis values after 90 days. ^b^P < 0.05 for baseline versus after 90 days within the group. Data are mean ± standard deviation. HOMA-IR, homeostasis model assessment of insulin resistance; HOMAβ, homeostasis model assessment of β-cell function.

**Table 6:** Effect of Iranian Propolis on lipid profile in T2DM patients after 90 days.

Variables	Propolis(n = 50)		Placebo(n = 44)		P-Value^a^
	Before	After	Before	After	
Triglycerides, mg/dl	162.8 ± 73.16	174.41 ± 101.91	164.52 ± 121.46	176.74 ± 83.59	0.91
Total cholesterol, mg/dl	149 ± 39.62	156.39 ± 42.98	153 ± 43.91	150.08 ± 38.96	0.488
HDL cholesterol, mg/dl	44.66 ± 8.69	48.91 ± 9.32^b^	43.98 ± 10.21	44.21 ± 9.24	0.024
LDL cholesterol, mg/dl	71.73 ± 29.48	70.59 ± 32.11	75.84 ± 26.2	73.67 ± 28.1	0.646
VLDL cholesterol, mg/dl	31.91 ± 13.81	37.28 ± 20.31	33.36 ± 27.27	32.71 ± 16.62	0.269

^a^Comparison of change between placebo and Iranian propolis values after 90 days. ^b^P < 0.05 for baseline versus after 90 days within the group. Data are mean ± standard deviation. HDL, high-density lipoprotein; LDL, low-density lipoprotein; VLDL, very low-density lipoprotein.

**Table 7:** Effect of Iranian propolis on Uric acid, renal and liver function tests in T2DM patients after 90 days.

Variables	Propolis(n = 50)		Placebo(n = 44)		P-Value^a^
	Before	After	Before	After	
BUN	12.57 ± 3.07	11.62 ± 2.64^b^	12.84 ± 3.59	12.3 ± 4.45	0.397
Cr	0.81 ± 0.2	0.85 ± 0.22	0.84 ± 0.3	0.96 ± 0.25	0.086
eGFR	110.91 ± 43.78	112.81 ± 99.95	114.31 ± 74.82	90.65 ± 25.87^b^	0.13
Uric acid	3.82 ± 1.19	3.63 ± 0.99	3.72 ± 1.54	3.8 ± 1.33	0.48
AST	27.42 ± 11.56	22.84 ± 6.18^b^	25.67 ± 9.98	24.92 ± 8.59	0.205
ALT	29.2 ± 26.26	22.45 ± 8.81^b^	26.91 ± 19.23	25.62 ± 12.97	0.193
ALP	240.68 ± 119.75	210.59 ± 61.01	280.55 ± 255.11	283.21 ± 226.18	0.044

^a^Comparison of change between placebo and Iranian propolis values after 90 days. ^b^P < 0.05 for baseline versus after 90 days within the group. Data are mean ± standard deviation. BUN, blood urea nitrogen; Cr, creatinine; eGFR, estimated glomerular filtration rate; AST, aspartate aminotransferase; ALT, alanine aminotransferase; ALP, alkalin phosphatase.

**Table 8:** Effect of Iranian propolis on level of hsCRP and cytokines in T2DM patients after 90 days.

Variables	Propolis(n = 50)		Placebo(n = 44)		P-Value^a^
	Before	After	Before	After	
hs-CRP, pg/ml	3589.74 ± ± 25.20.96	3024.12 ± 2368.5	3925.92 ± 2546.47	6033.02 ± 2350.87^b^	0.001
TNF, pg/ml	122.98 ± 115.44	85.69 ± 84.7^b^	130.24 ± 11.99	146.41 ± 141.03^b^	0.000
IL6, pg/ml	32.88 ± 44.61	24.4 ± 23.68	23.05 ± 22.03	27.02 ± 23.82	0.405
IL1B, pg/ml	34.84 ± 32.74	29.3 ± 25.66^b^	23.72 ± 13.42	25.27 ± 14.25	0.373

^a^Comparison of change between placebo and Iranian propolis values after 90 days. ^b^P < 0.05 for baseline versus after 90 days within the group. Data are mean ± standard deviation. TNF-α, tumor necrosis factor-α; hs-CRP, high sensitivity C-reactive protein; IL-1β, interleukin-1β; IL-6, interleukin-6.

## Results

### Demographic and laboratory information of patients at baseline

**Figure 1:**
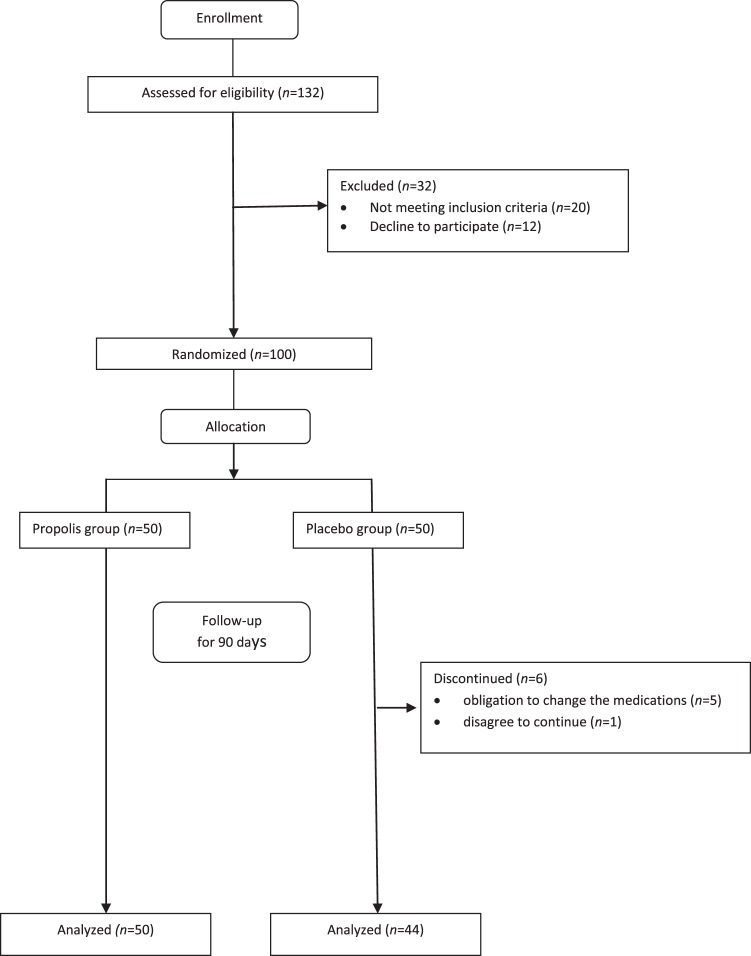
Enrollment and outcomes of the study.

Between September 2017 and March 2018, 100 patients were enrolled in this study. They were randomized into either group: administration with Iranian propolis (n = 50) or placebo (n = 50). Six patients from the placebo group withdrew during the study; five of the six patients experienced a change in their hyperglycemia and were obliged to change their medications. One of the patients also left for the personal reasons. Ninety-four patients completed the study (Fig. 1). Table 1. outlines the demographics and general characteristics of these 94 patients are s in Table 1. The mean age, the duration of diabetes, male and female proportion, the mean systolic/diastolic blood pressure, body weight, BMI and physical activity were comparable between both groups. Oral hypoglycemic drugs used at the start of the study had no statistical significance between the two groups.

Furthermore, baseline parameters of glucose metabolism, renal and liver function, lipid metabolism and inflammatory cytokines also showed no significant differences. (Table 2)

### Dietary intake of energy and nutrients

As shown in Table 3, there was no significant difference in dietary intake of energy between the Iranian propolis and placebo groups. The nutrients intake was very similar with the exception of selenium and riboflavin. The patients in the Iranian propolis group had significantly lower daily dietary intake of selenium and riboflavin than those in the placebo group; 25% and 13.26% respectively.

No diet changes or additional physical activities were assigned in an interview after finishing the study. Measurements of both groups, at the end of the study, demonstrated no statistically significant change in body weight and BMI (Table 4).

### Effect of Iranian propolis on glucose metabolism

HbA1C, FBS, 2hpp blood sugar (2hpp BS) and insulin were measured on day 0 and day 90 as shown in Table 5. At end of the intervention, the mean HbA1C, 2hpp BS and insulin significantly decreased in the propolis group compared with the placebo group by 8% (p = 0.006), 28.6% (p < 0.0001) and 50.8 (p < 0.0001) respectively. There was no significant difference in FBS between the two groups. After consuming Iranian propolis HOMA-IR and HOMA-β were significantly decreased by 46.6% and 45.8% respectively compared with the placebo group (p < 0.0001).

On day 90 the propolis group had a mean of 0.98% reduction in HbA1C from 8.65 ± 1.24 to 7.67 ± 1.27 (p < 0.001) and a 45% reduction in insulin levels from 14.03 ± 15.43 to 7.61 ± 6.69 (p = 0.001) compared to the data prior to propolis administration. Also, the placebo group showed a 32.93% increase in 2hpp BS from 168 ± 05 to 223.39 ± 82.04 by the end of the study (p < 0.0001).

### Effect of Iranian propolis on lipid profile

The effects of Iranian propolis on blood lipid profile in T2DM patients are given in Table 6. After the administration of propolis, the mean HDL-C was significantly increased when compared to the placebo group by 10.6% (*p* = *0*.*024)*. The propolis group had a 9.5% increase in HDL-C at day 90 from 44.66 ± 8.69 to 48.91 ± 9.32 mg/dl (*p* < *0*.*0001)* compared to the baseline.

There were no statistically significant differences in total cholesterol, LDL-C, TG and VLDL in both groups (*p* > *0*.*05)*.

### Effect of Iranian propolis on uric acid, renal and liver function tests

As shown in Table 7 serum uric acid, BUN, Cr, eGFR, ALT, AST and alkaline phosphatase (ALP) did not significantly change in the both groups. However, it is notable that the BUN, AST and ALT levels in the propolis group significantly deceased after 90 days compared to the baseline by 7.5% (from 12.57 ± 3.07 to 11.62 ± 2.64; p = 0.043), 16.7% (from 27.42 ± 11.56 to 22.45 ± 8.81; p = 0.01) and 23% (from 29.2 ± 26.26 to 22.45 ± 8.81; p = 0.01) respectively. Furthermore, eGFR decreased by 20.7% (from 114.31 ± 74.82 to 90.65 ± 25.87; p < 0.0001) within the placebo group by day 90 compared to baseline, while the initial level was maintained by the Iranian propolis group. (p = 0.29).

### Effect of Iranian propolis on hs-CRP and cytokines

As seen in Table 8, after the administration of Iranian propolis, serum hs-CRP and TNF-α significantly decreased by 60.43% (p = 0.001) and 49.6% (p < 0.0001) respectively, in comparison to the placebo group. However, no significant difference was noted for serum IL-1β and IL-6 levels between the two groups.

At the day 90 the propolis group had a mean of 30% reduction in serum TNF-α from 122.98 ± 115.44 to 85.69 ± 84.7 pg/ml (p = 0.003) and a 16% reduction in serum IL1-β from 34.84 ± 32.74 to 29.3 ± 25.66 pg/ml (p = 0.044) compared to the baseline. In the placebo group, there was a 53.7% increase in hs-CRP from 3925.92 ± 2546.47 to 6033.02 ± 2350.87 pg/ml (p = 0.007) and a 12.4% increase in TNF-α from 130.24 ± 11.99 to 146.41 ± 141.03 pg/ml (p < 0.001).

## Discussion

Diabetes Mellitus (DM) is a prevalent chronic disorder characterized by an elevated blood glucose concentration and recently it has been shown that oxidative stress and free radicals as well as inflammatory cytokines are determinant in its pathogenesis and complications. Propolis has a strong antioxidant and free radical scavenging effect as well as significant anti inflammation properties which can offers a promising therapeutic value in treatment or prevention of T2DM progression^17^.

The present study revealed that consumption of Iranian propolis for 90 days can significantly decrease the serum levels of HbA1C, insulin and 2-hpp glucose and enhance the insulin sensitivity in T2DM patients. Many studies showed that propolis has decreased blood glucose, insulin and HbA1C levels and increased insulin sensitivity in T2DM models^6,50,56–58^. It has been suggested that the glycemic control achieved by propolis treatment might be as a result of reducing intestinal absorption of carbohydrate, increasing the level of glycolysis and utilization of glucose in the liver, triggering glucose uptake by peripheral tissue like skeletal muscle cells by activating insulin-sensitive glucose transporter, and inhibition of its release in circulation from the liver^48,59,60^. Zhang *et al*., reported that propolis extract possesses much stronger inhibitory effects on α-glycosidase and intestinal sucrase compared to synthetic α-glycosidase inhibitor such as acarbose^49^. Also Matsui *et al*., demonstrated that propolis exerts its anti-hyperglycemic effect through the inhibition of glucose production from dietary carbohydrates and strongly suggested the use of propolis for controlling or delaying the postprandial glucose rise and improving insulin resistance^48^. For the first time we have demonstrated that propolis can significantly improve glucose metabolism in T2DM patients in a double-blind randomized clinical trial. Similar clinical studies recently reported that Chinese and Brazilian propolis did not significantly improve glucose profile^52–54^, although they did not measure postprandial glucose.

Every 1% reduction in HbA1C results in about 21% reduction in all complications of diabetes^61,62^. In this study Iranian propolis reduced HbA1C by 0.98%, which is then effective in the prevention of diabetes complications. This result is compatible with the findings reported by Murata *et al*., who demonstrated that propolis mixed with mulberry leaf extract showed an average reduction of 0.8% in HbA1C levels in T2DM patients close to that of acarbose^61^.

Some studies have shown that propolis improves insulin sensitivity and reduces insulin levels in T2DM^17,57,58,63^. Insulin resistance has been linked to the development of cardiovascular diseases^58^. In this study, HOMA-IR and HOMAβ were calculated to assess the effect of Iranian propolis on insulin resistance in T2DM patients. Our study revealed that propolis supplementation decreased the levels of blood postprandial glucose and serum insulin levels, as well as, decreased insulin resistance in T2DM patients.

Diabetes mellitus is almost always associated with the deterioration of plasma lipoprotein profile and, therefore, it can increase atherogenesis in human diabetic patients^50^. Propolis enhances liver protein expression of the ATP-binding cassette transporters, which are related to cholesterol efflux from peripheral tissue and HDL particle formation^64^. Mujica *et al*., has observed that 90 days of propolis consumption, in a randomized clinical trial, caused significant increase in HDL-C levels in the general population^65^. In the present study, Iranian propolis supplementation increased HDL-C in T2DM patients. It is known that HDL-C is an important lipoparticle which provides protection against cardiovascular disease, prevents LDL oxidation, and neutralizes atherogenic effects in arterial walls^65^.

Chronic mild elevations of transaminases are frequently found in patients with T2DM and are associated with insulin resistance and are due to oxidative stress^66^. The consumption of antidiabetic agents decrease ALT levels as more stable blood glucose levels are maintained^66^. Supplementation of Iranian propolis was found to significantly decrease the concentration of liver transaminase (ALT and AST) (p < 0.05; Table 7). Celik *et al*., reported that administration of caffeic acid phenethyl ester (CAPE), one of the active components of propolis, exhibited hepatoprotective effects, and reduced the levels of transaminase in diabetic rats^43^.

An intake of 1000 mg of Iranian propolis, for 90 days, maintained baseline eGFR levels and reduced BUN levels significantly (Table 7). In contrast, the eGFR levels in the placebo group significantly decreased post 90 days. These observations indicate that 90 days of propolis supplementation prevents deterioration of renal function in diabetic patients. Interestingly, the same result was reported by Fudaka *et al*., where the effects of Brazilian green propolis on eGFR levels in patient with T2DM were considered^54^. Abo-salem *et al*., observed that, in diabetic rats, propolis significantly declines BUN concentrations, reverting them back to normal levels, and preventing diabetic nephropathy^45^.

High levels of pro-inflammatory cytokines (IL1, IL6 and TNF-α) and hs-CRP are strongly associated with chronic inflammation and oxidative stress in the pathogenesis of T2DM^52,67^. C-reactive protein (CRP) is a marker for systemic inflammation, has a significant role in the pathogenesis of atherosclerotic lesions^49^ and may predict T2DM development and its cardiovascular complications^68,69^.

Propolis has strong anti-inflammation properties^70^ and also can directly decrease the levels of pro-inflammatory cytokines^16,19,71^. While most studies agree that propolis has a positive impact on the inflammation processes, and that it significantly decreases the levels of TNF-α, its effects on IL-1β and IL6 levels has been controversial^19,72–74^.

In this study, we observed that after the administration of Iranian propolis, in T2DM patients, serum hs-CRP and TNF-α levels were significantly decreased. However, no difference was noted for the levels of serum IL-1β and IL6. We also found that the propolis group had a mean reduction of 16% in serum IL1-β compared to the baseline (Table 8). Zaho *et al*., reported the administration of Brazilian green propolis resulted in a considerable reduction in serum TNF-α levels, in T2DM patients, whereas, serum IL-1β and IL-6 were significantly increased. The authors noted that the pro-inflammatory effects of IL-1β production might be prevented by the anti-inflammatory effects of IL6, hence, the overall result of Brazilian green propolis on chronic inflammation is positive in T2DM patients^52^. Fukuda *et al*. showed no significant alternation in IL-6 by the administration of Brazilian green propolis in T2DM although TNF-α in the placebo group revealed a propensity to increase^54^.

Se and riboflavin baseline daily dietary intake were both significantly higher in placebo group than propolis in our study. However, no evidence has been presented to prove that these nutrients can induce glucose intolerance or insulin resistance^75,76^.

A limitation encountered in our study was due to the fact that the effective components and their doses, within the capsules, were not identified. Sforcin *et al*., suggested that the effects of propolis may be the result of several components and not in isolation of one or a few compounds^77^.

## Conclusion

In summary, this study demonstrated that Iranian propolis has beneficial effects on reducing post prandial blood glucose, serum insulin, insulin resistance and inflammatory cytokines. Furthermore, Iranian propolis can also prevent liver and renal dysfunction, as well as, elevating HDL-C concentrations in patients with T2DM.

## Supplementary information

project summary

consort 2010
