# Supplementary material for: The Effect of Iranian Propolis on Glucose Metabolism, Lipid Profile, Insulin Resistance, Renal Function and Inflammatory Biomarkers in Patients with Type 2 Diabetes Mellitus: A Randomized Double-Blind Clinical Trial
Source: Sci Rep. 2019 May 13;9:7289. doi: 10.1038/s41598-019-43838-8 (PMC6514000; doi:10.1038/s41598-019-43838-8)
Supplement: Supplementary file 1 — project summary [file 41598_2019_43838_MOESM1_ESM.docx]

**Project summary**

***Introduction:*** Propolis is a natural product with many biological properties including hypoglycemic activity and modulating lipid profile. The present study was designed to evaluate the effect of Iranian propolis extract on glucose metabolism, Lipid profile, Insulin resistance, renal and liver function as well as inflammatory biomarkers in patients with type 2 diabetes mellitus (T2DM).

***Methods:*** A double-blinde, Placebo-controlled clinical trial was conducted. In 90 days study, recruited T2DM patients were randomly divided into an Iranian propolis group (1000 mg/day) (n=50) and a placebo group (n=44).

***Results:*** At the end of the study, the serum levels of glycosylated hemoglobin (HbA1c), 2-hour post prandial (2hpp), insulin, homeostasis model assessment-insulin resistance (HOMA-IR), homeostasis model assessment of β-cell function (HOMA-β), High sensitive C-reactive protein (hs-CRP), tumor necrosis factor-α (TNF-α) significantly decreased and serum HDL-C was significantly increased in the propolis group compared with the placebo group. In the propolis group, serum liver transaminase (ALT and AST) and blood urea nitrogen (BUN) concentrations significantly decreased after 90 days.

***Conclusion:*** Iranian propolis has beneficial effects on reducing post prandial blood glucose, serum insulin, insulin resistance and inflammatory cytokines also it can prevent the liver and renal dysfunction as well as can elevate HDL-C concentrations in T2DM patients.

**General information**

- The Effect of Iranian Propolis on Glucose Metabolism, Lipid Profile, Insulin Resistance, Renal Function and Inflammatory Biomarkers in Patients with Type 2 Diabetes Mellitus: A Randomized Double Blind Clinical Trial. Sep 2017
- Name and address of Funder: Ahvaz Jundishapur University of Medical Sciences, Golestan, Ahvaz, Iran
- Name of the investigator who conducted the research: Dr. Mehrnoosh Zakerkish ; Assistant Professor, Endocrinologist; Diabetes Research Center, Ahvaz Jundishapur University of Medical Sciences, Ahvaz, Iran; +986133369539
- Name and address of clinical laboratory: Diabetes Research Center, Ahvaz Jundishapur University of Medical Sciences, Ahvaz, Iran
- Name and addreass of other institution: Shahdine Golha, Isfahan, Iran

**Rationale & background information**

Diabetes Mellitus (DM) is a common chronic disorder characterized by an elevated blood glucose concentration which is associated with absolute (type 1) or relative (type 2) deficiencies in insulin secretion and/or action. These conditions are summarized as T1DM and T2DM, respectively (1).

The prevalence of diagnosed diabetes is dramatically increasing worldwide. According to the International Diabetes Federation the number of diabetic patients (most of them with T2DM) will be risen from 366 million in 2011 to 439 million by 2030 that cause mainly by the increasing western sedentary lifestyle and obesity (2).

Poorly controlled type 2 diabetes is associated to microvascular (retinopathy and nephropathy), macrovascular, and non-vascular (neuropathy) complications which lead to poor prognosis and a significant decrease in life expectancy (3). Thus the death rate among diabetic patients is about twice in compare with general population and also by 2015 DM was the seventh leading cause of death in the United States (4). Besides the elevated rate of morbidity and mortality, economically the total cost of care of patients with T2DM and its complications is very high (5).

Previous studies have demonstrated that diabetes increases the production of inflammatory mediators, such as tumor necrosis factor-α (TNF-α), interleukin-6 (IL-6) and interleukin 1 (IL-1) which are the main cytokines involved in diabetes pathogenesis (6-10). Therefore, most interest has been focused on the development of alternative medicinal food such as natural bioactive products that are able to control blood glucose concentration and decrease the risk of complications (11).

Since oxidative stress that caused by persistent hyperglycemia play a main role in the progression of the DM and its complication (12, 13), recently many works considered the use of natural antioxidant bioactive compounds as new adjunctive pharmaceuticals for reducing T2DM complications (14).

Propolis is a natural resinous hive product that honeybees (*Apis mellifera L*.) collect from various plants sources and mix with their salivary gland enzymes and wax to use it for sealing holes in their honeycombs, smoothing out internal walls and protecting the entrance against intruders (15).

Propolis has a long history of being used in folk medicine dates back to ancient time, at least to 300 BC, in many countries and since then has been largely utilized as a medicine for its distinctive biological properties specially antioxidant activity in various dosing forms (16-18).

Propolis chemical composition is very complex and depends on the phytogeographic characteristics like vegetation, season, and environmental condition of the site of collection since bees select different plants in different habitats to produce propolis (14, 19). This aspect makes universal standardization of propolis somehow impossible (20). Over 300 active constituents have been isolated from propolis; In general they are mostly a mixture of phenols (e.g., flavonoid, polyphenol, and aromatic compounds), terpenes, amino acids, vitamins, sugars, and elements (21). Therefore, since plant origin and the region where propolis is collected have a significant influence on its composition, the country of origin should be mentioned (e.g., Iranian propolis). The most recent study has reported that Iranian propolis is rich in flavonoids, phenolic and terpene compounds similar to Cuban, Brazilian and Egyptian propolis (22). Chemical analysis of Iranian propolis used for this study revealed that the major components are flavonoids and phenolic acid esters. However, it is well known that despite of chemical composition differences in the samples of different geographical origins, they usually show similar pharmacological properties to some extent (15).

Propolis biological and pharmacological properties recently have attracted researcher’s interest and many studies have demonstrated it possesses antimicrobial (23-25), immunomudulatory (26), antitumor (27-29), anti-inflammatory (30-32), antioxidant (33-35), antiviral (36, 37), antifungal (38, 39) as well as hepatoprotective, nephroprotective and pancreatoprotective activities (40-44). According to recent studies, propolis also has hypoglycemic activity and may have some positive effect on diabetic complications as well as modulating the metabolism of blood lipid leading to decrease lipid peroxidation and scavenge the free radicals (5, 45-48). The broad spectrum of biological activity beside the long history of use and safety profile make propolis to be considered as a potential clinically useful drug (14, 17).

To the best of our knowledge, the clinical trials concerning propolis efficacy on T2DM are few and with no uniform criteria and their results are significantly controversial (49-51). Thereby, further clinical studies should be performed to clarify the efficacy of propolis in the treatment of T2DM.

**References (of literature cited in preceding sections)**

1. King H, Aubert RE, Herman WH. Global burden of diabetes, 1995-2025: prevalence, numerical estimates, and projections. Diabetes Care. 1998;21(9):1414-31.

2. Guariguata L, Whiting DR, Hambleton I, Beagley J, Linnenkamp U, Shaw JE. Global estimates of diabetes prevalence for 2013 and projections for 2035. Diabetes Res Clin Pract. 2014;103(2):137-49.

3. Intensive blood-glucose control with sulphonylureas or insulin compared with conventonal treatment and risk of complications in patients with type 2 diabetes (UKPDS 33). In: group UPDSU, editor.: Lancet; 1998 p. 837-53.

4. Centers for Disease Control and Prevention. National Diabetes Statistics Report, 2017. Available at <https://www.cdc.gov/diabetes/pdfs/data/statistics/national-diabetes-statistitics-report.pdf> [Internet]. 2017.

5. Li Y, Chen M, Xuan H, Hu F. Effects of encapsulated propolis on blood glycemic control, lipid metabolism, and insulin resistance in type 2 diabetes mellitus rats. Evid Based Complement Alternat Med. 2012;2012:981896.

6. Alexandraki K, Piperi C, Kalofoutis C, Singh J, Alaveras A, Kalofoutis A. Inflammatory process in type 2 diabetes: The role of cytokines. Ann N Y Acad Sci. 2006;1084:89-117.

7. Maedler K, Sergeev P, Ris F, Oberholzer J, Joller-Jemelka HI, Spinas GA, et al. Glucose-induced beta cell production of IL-1beta contributes to glucotoxicity in human pancreatic islets. J Clin Invest. 2002;110(6):851-60.

8. Kristiansen OP, Mandrup-Poulsen T. Interleukin-6 and diabetes: the good, the bad, or the indifferent? Diabetes. 2005;54 Suppl 2:S114-24.

9. Rabinovitch A, Sumoski W, Rajotte RV, Warnock GL. Cytotoxic effects of cytokines on human pancreatic islet cells in monolayer culture. J Clin Endocrinol Metab. 1990;71(1):152-6.

10. Ruan H, Lodish HF. Insulin resistance in adipose tissue: direct and indirect effects of tumor necrosis factor-alpha. Cytokine Growth Factor Rev. 2003;14(5):447-55.

11. Davi G, Santilli F, Patrono C. Nutraceuticals in diabetes and metabolic syndrome. Cardiovasc Ther. 2010;28(4):216-26.

12. Mercuri F, Quagliaro L, Ceriello A. Oxidative stress evaluation in diabetes. Diabetes Technol Ther. 2000;2(4):589-600.

13. West IC. Radicals and oxidative stress in diabetes. Diabet Med. 2000;17(3):171-80.

14. Sforcin JM, Bankova V. Propolis: is there a potential for the development of new drugs? J Ethnopharmacol. 2011;133(2):253-60.

15. Silva-Carvalho R, Baltazar F, Almeida-Aguiar C. Propolis: A Complex Natural Product with a Plethora of Biological Activities That Can Be Explored for Drug Development. Evid Based Complement Alternat Med. 2015;2015:206439.

16. Castaldo S, Capasso F. Propolis, an old remedy used in modern medicine. Fitoterapia. 2002;73 Suppl 1:S1-6.

17. Burdock GA. Review of the biological properties and toxicity of bee propolis (propolis). Food Chem Toxicol. 1998;36(4):347-63.

18. Khalil ML. Biological activity of bee propolis in health and disease. Asian Pac J Cancer Prev. 2006;7(1):22-31.

19. Toreti VC, Sato HH, Pastore GM, Park YK. Recent progress of propolis for its biological and chemical compositions and its botanical origin. Evid Based Complement Alternat Med. 2013;2013:697390.

20. Bankova V. Chemical diversity of propolis and the problem of standardization. J Ethnopharmacol. 2005;100(1-2):114-7.

21. Bankova V, Popova M, Trusheva B. Propolis volatile compounds: chemical diversity and biological activity: a review. Chem Cent J. 2014;8:28.

22. Afrouzan H, Tahghighi A, Zakeri S, Es-haghi A. Chemical Composition and Antimicrobial Activities of Iranian Propolis. Iran Biomed J. 2018;22(1):50-65.

23. Massaro CF, Simpson JB, Powell D, Brooks P. Chemical composition and antimicrobial activity of honeybee (Apis mellifera ligustica) propolis from subtropical eastern Australia. Naturwissenschaften. 2015;102(11-12):68.

24. Nina N, Quispe C, Jimenez-Aspee F, Theoduloz C, Feresin GE, Lima B, et al. Antibacterial Activity, Antioxidant Effect and Chemical Composition of Propolis from the Region del Maule, Central Chile. Molecules. 2015;20(10):18144-67.

25. Trusheva B, Todorov I, Ninova M, Najdenski H, Daneshmand A, Bankova V. Antibacterial mono- and sesquiterpene esters of benzoic acids from Iranian propolis. Chem Cent J. 2010;4:8.

26. Sforcin JM. Propolis and the immune system: a review. J Ethnopharmacol. 2007;113(1):1-14.

27. Demir S, Aliyazicioglu Y, Turan I, Misir S, Mentese A, Yaman SO, et al. Antiproliferative and proapoptotic activity of Turkish propolis on human lung cancer cell line. Nutr Cancer. 2016;68(1):165-72.

28. Salim EI, Abd El-Magid AD, Farara KM, Maria DS. Antitumoral and Antioxidant Potential of Egyptian Propolis Against the PC3 Prostate Cancer Cell Line. Asian Pac J Cancer Prev. 2015;16(17):7641-51.

29. Xuan H, Li Z, Yan H, Sang Q, Wang K, He Q, et al. Antitumor Activity of Chinese Propolis in Human Breast Cancer MCF-7 and MDA-MB-231 Cells. Evid Based Complement Alternat Med. 2014;2014:280120.

30. Bueno-Silva B, Kawamoto D, Ando-Suguimoto ES, Alencar SM, Rosalen PL, Mayer MP. Brazilian Red Propolis Attenuates Inflammatory Signaling Cascade in LPS-Activated Macrophages. PLoS One. 2015;10(12):e0144954.

31. Hu F, Hepburn HR, Li Y, Chen M, Radloff SE, Daya S. Effects of ethanol and water extracts of propolis (bee glue) on acute inflammatory animal models. J Ethnopharmacol. 2005;100(3):276-83.

32. Franchin M, Freires IA, Lazarini JG, Nani BD, da Cunha MG, Colon DF, et al. The use of Brazilian propolis for discovery and development of novel anti-inflammatory drugs. Eur J Med Chem. 2018;153:49-55.

33. Zhang J, Shen X, Wang K, Cao X, Zhang C, Zheng H, et al. Antioxidant activities and molecular mechanisms of the ethanol extracts of Baccharis propolis and Eucalyptus propolis in RAW64.7 cells. Pharm Biol. 2016;54(10):2220-35.

34. Wali AF, Avula B, Ali Z, Khan IA, Mushtaq A, Rehman MU, et al. Antioxidant, Hepatoprotective Potential and Chemical Profiling of Propolis Ethanolic Extract from Kashmir Himalaya Region Using UHPLC-DAD-QToF-MS. Biomed Res Int. 2015;2015:393462.

35. de Mendonca IC, Porto IC, do Nascimento TG, de Souza NS, Oliveira JM, Arruda RE, et al. Brazilian red propolis: phytochemical screening, antioxidant activity and effect against cancer cells. BMC Complement Altern Med. 2015;15:357.

36. Gekker S Hu, Spivak M, Lokensgard JR, Peterson PK. Anti-HI-1 activity of propolis in CD4 lymphocyte and microglial cell cultures. Journal of ethnopharmacology. 2005;102(2):158-63.

37. Sartori G, Pesarico AP, Pinton S. Protective effect of brown Brazilian propolis against acute vaginal lesions caused by herpes simplex virus type 2 in mice involvement of antioxidant and anti-inflammatory mechanisms. Cell Biochemistry and Function. 2012;30(1):1-10.

38. Freires IA, Queiroz VC, Furletti VF, Ikegaki M, de Alencar SM, Duarte MC, et al. Chemical composition and antifungal potential of Brazilian propolis against Candida spp. J Mycol Med. 2016;26(2):122-32.

39. Mutlu Sariguzel F, Berk E, Koc AN, Sav H, Demir G. Antifungal Activity of Propolis Against Yeasts Isolated From Blood Culture: In Vitro Evaluation. J Clin Lab Anal. 2016.

40. Tolba MF, Azab SS, Khalifa AE, Abdel-Rahman SZ, Abdel-Naim AB. Caffeic acid phenethyl ester, a promising component of propolis with a plethora of biological activities: a review on its anti-inflammatory, neuroprotective, hepatoprotective, and cardioprotective effects. IUBMB Life. 2013;65(8):699-709.

41. Bhadauria M, Nirala SK, Shukla S. Duration-dependent hepatoprotective effects of propolis extract against carbon tetrachloride-induced acute liver damage in rats. Adv Ther. 2007;24(5):1136-45.

42. Abo-Salem OM, El-Edel RH, Harisa GE, El-Halawany N, Ghonaim MM. Experimental diabetic nephropathy can be prevented by propolis: Effect on metabolic disturbances and renal oxidative parameters. Pak J Pharm Sci. 2009;22(2):205-10.

43. Babatunde IR, Abdulbasit A, Oladayo MI, Olasile OI, Olamide FR, Gbolahan BW. Hepatoprotective and Pancreatoprotective Properties of the Ethanolic Extract of Nigerian Propolis. J Intercult Ethnopharmacol. 2015;4(2):102-8.

44. Orsolic N, Sirovina D, Koncic MZ, Lackovic G, Gregorovic G. Effect of Croatian propolis on diabetic nephropathy and liver toxicity in mice. BMC Complement Altern Med. 2012;12:117.

45. Matsui T, Ebuchi S, Fujise T. Strong antihyperglycemic effects of water-souluble fraction of brazillian propolis and its bioactive constituent, 3,4,5-tri-o-caffeoylquinic acid. biol Pharm ull. 2004;27(11):1797-803.

46. Zhang H, Wang G, Beta T, Dong J. Inhibitory properties of aqueous ethanol extracts of propolis on alpha-glucosidase. Evid Based Complement Alternat Med. 2015;2015:587383.

47. Oladayo MI. Nigerian propolis improves blood glucose, glycated hemoglobin A1c, very low-density lipoprotein, and high-density lipoprotein levels in rat models of diabetes. J Intercult Ethnopharmacol. 2016;5(3):233-8.

48. Fuliang HU, Hepburn HR, Xuan H, Chen M, Daya S, Radloff SE. Effects of propolis on blood glucose, blood lipid and free radicals in rats with diabetes mellitus. Pharmacol Res. 2005;51(2):147-52.

49. Zhao L, Pu L, Wei J, Li J, Wu J, Xin Z, et al. Brazilian Green Propolis Improves Antioxidant Function in Patients with Type 2 Diabetes Mellitus. Int J Environ Res Public Health. 2016;13(5).

50. Gao W, Pu L, Wei J, Yao Z, Wang Y, Shi T, et al. Serum Antioxidant Parameters are Significantly Increased in Patients with Type 2 Diabetes Mellitus after Consumption of Chinese Propolis: A Randomized Controlled Trial Based on Fasting Serum Glucose Level. Diabetes Ther. 2018;9(1):101-11.

51. Fukuda T, Fukui M, Tanaka M, Senmaru T, Iwase H, Yamazaki M, et al. Effect of Brazilian green propolis in patients with type 2 diabetes: A double-blind randomized placebo-controlled study. Biomed Rep. 2015;3(3):355-60.

52. American Diabetes A. Diagnosis and classification of diabetes mellitus. Diabetes Care. 2010;33 Suppl 1:S62-9.

53. Popova M, Bankova V, Butovska D, Petkov V, Nikolova-Damyanova B, Sabatini AG, et al. Validated methods for the quantification of biologically active constituents of poplar-type propolis. Phytochem Anal. 2004;15(4):235-40.

54. sartori D, Kawakami c, Orsatti C, Sforcin J. propolis effect on streptozotocin-induced diabetic rats. journal of venom animal toxin incl trop disease. 2009;15(1):93-102.

55. Sameni HR, Ramhormozi P, Bandegi AR, Taherian AA, Mirmohammadkhani M, Safari M. Effects of ethanol extract of propolis on histopathological changes and anti-oxidant defense of kidney in a rat model for type 1 diabetes mellitus. J Diabetes Investig. 2016;7(4):506-13.

56. El-Sayed el SM, Abo-Salem OM, Aly HA, Mansour AM. Potential antidiabetic and hypolipidemic effects of propolis extract in streptozotocin-induced diabetic rats. Pak J Pharm Sci. 2009;22(2):168-74.

57. Major CD, Gao ZY, Wolf BA. Activation of the sphingomyelinase/ceramide signal transduction pathway in insulin-secreting beta-cells: role in cytokine-induced beta-cell death. Diabetes. 1999;48(7):1372-80.

58. El-kherbawy G, Noub S.G, Abd El-Aziz H.M, zaki S.A. Effect of cinnamon, propolis, or their combination on blood glucose, body weight, feed efficiency ratio and relative organs' weights in rats with diabetes mellitus. 2009.

59. Kitamura H, Naoe Y, Kimura S, Miyamoto T, Okamoto S, Toda C, et al. Beneficial effects of Brazilian propolis on type 2 diabetes in ob/ob mice: Possible involvement of immune cells in mesenteric adipose tissue. Adipocyte. 2013;2(4):227-36.

60. Aoi W, Hosogi S, Niisato N, Yokoyama N, Hayata H, Miyazaki H, et al. Improvement of insulin resistance, blood pressure and interstitial pH in early developmental stage of insulin resistance in OLETF rats by intake of propolis extracts. Biochem Biophys Res Commun. 2013;432(4):650-3.

61. Al-Hariri M, Eldin TG, Abu-Hozaifa B, Elnour A. Glycemic control and anti-osteopathic effect of propolis in diabetic rats. Diabetes Metab Syndr Obes. 2011;4:377-84.

62. Murata K, Yatsunami K, Fukuda E, Onodera S, Mizukami O, Hoshino G, et al. Antihyperglycemic effects of propolis mixed with mulberry leaf extract on patients with type 2 diabetes. Altern Ther Health Med. 2004;10(3):78-9.

63. Zamami Y, Takatori S, Koyama T, Goda M, Iwatani Y, Doi S, et al. [Effect of propolis on insulin resistance in fructose-drinking rats]. Yakugaku Zasshi. 2007;127(12):2065-73.

64. Al Ghamdi AA, Badr G, Hozzein WN, Allam A, Al-Waili NS, Al-Wadaan MA, et al. Oral supplementation of diabetic mice with propolis restores the proliferation capacity and chemotaxis of B and T lymphocytes towards CCL21 and CXCL12 by modulating the lipid profile, the pro-inflammatory cytokine levels and oxidative stress. BMC Immunol. 2015;16:54.

65. Yu Y, Si Y, Song G, Luo T, Wang J, Qin S. Ethanolic extract of propolis promotes reverse cholesterol transport and the expression of ATP-binding cassette transporter A1 and G1 in mice. Lipids. 2011;46(9):805-11.

66. Mujica V, Orrego R, Perez J, Romero P, Ovalle P, Zuniga-Hernandez J, et al. The Role of Propolis in Oxidative Stress and Lipid Metabolism: A Randomized Controlled Trial. Evid Based Complement Alternat Med. 2017;2017:4272940.

67. EH H. Elevated liver function tests in type 2 diabets. clinical diabets. 2005;23:115-9.

68. Freires I, De Alencar SM, Rosalen PL. A pharmacological perspective on the use of Brazilian red propolis and its isolated compounds against human disease. European Journal of Medicinal Chemistry. 2016.

69. Pradhan AD, Manson JE, Rifai N, Buring JE, PM R. C-reactive protein, interleukin 6, and risk of developing type 2 diabetes mellitus. JAMA. 2001;286:286-334.

70. Navarro-Gonzalez JF, Mora-Fernandez C. The role of inflammatory cytokines in diabetic nephropathy. J Am Soc Nephrol. 2008;19(3):433-42.

71. Baig M, Sarwari K, sabeer H. Study of serum hs-CRP in type 2 diabetic patients. International journal of basic and applied medical sciences. 2013;3:235-40.

72. Funakoshi-Tago M, Okamoto K, Izumi R, Tago K, Yanagisawa K, Narukawa Y, et al. Anti-inflammatory activity of flavonoids in Nepalese propolis is attributed to inhibition of the IL-33 signaling pathway. Int Immunopharmacol. 2015;25(1):189-98.

73. Orsatti CL, Missima F, Pagliarone AC, Bachiega TF, Bufalo MC, Araujo JP, Jr., et al. Propolis immunomodulatory action in vivo on Toll-like receptors 2 and 4 expression and on pro-inflammatory cytokines production in mice. Phytother Res. 2010;24(8):1141-6.

74. Bachiega TF, Orsatti CL, Pagliarone AC, Sforcin JM. The effects of propolis and its isolated compounds on cytokine production by murine macrophages. Phytother Res. 2012;26(9):1308-13.

**Study goals and objectives**

We evaluate the effect of Iranian propolis extract on glucose metabolism, Lipid profile, Insulin resistance, renal and liver function as well as inflammatory biomarkers in patients with T2DM

**Study Design**

Enrolled participants. Patients with T2DM were screened and enrolled from the Department of Endocrinology, Golestan hospital, Ahvaz, Iran. T2DM was diagnosed in accordance with the criteria of the American Diabetes Mellitus Associations (52). Patients were aged between 35-85 years, receiving treatment with oral hypoglycemic agents.

Excluded participants. The patients treated with insulin; severe renal dysfunction [estimated glumerular filtration rate (eGFR) <30 ml/min/1.73m2]; severe hepatic dysfunction (aspartate aminotransferase (AST) >100 U/I) or alanine aminotransferase (ALT) >100 U/I); serious cardiovascular and hematological disease; diabetes diagnosed history >10 years; any kind of allergies and women in pregnancy or lactation.

**Methodology**

Iranian propolis capsules used in this study were provided from Shahdine Golha Co. (Isfahan, Iran) that were collected from bee hives located in different parts of the Eastern Azarbayejan province during the fall season and veriﬁed by an agricultural organization. Each capsule contains 500 mg Iranian propolis.

Iranian propolis is a kind of poplar type propolis, so chemical proﬁle should be characterized by the three parameters: total ﬂavones/ﬂavonols, ﬂavanones/dihydroﬂavonols, and phenolic compounds content which are used as a measure for the amount of active principles (20). The spectrophotometric assay based on the formation of aluminium chloride complex was applied for quantiﬁcation of total ﬂavones/ﬂavonols (53). For the measurement of amount of ﬂavanones/ dihydroﬂavonols colorimetric method with DNP (2, 4 dinitrophenylhydrazine) was used and total phenolic compounds content was measured by the Folin–Ciocalteu procedure (53). According to investigation for “typical poplar sample” and based on statistics these amounts should be as following; ﬂavones/ﬂavonols: 8 ± 4%, ﬂavanones/dihydroﬂavonols: 6 ± 2%, total phenolic compounds: 28 ± 9% (20). Our samples were in the range (total flavones and flavonols: 8.4%, total flavanones and dihydroflavonols: 4.6% and total phenolic compounds: 28%).

The present study was a randomized, double blind study so for allocation of the patients, the “Random Allocation” Software was used. The placebo served as a references drug for comparison. The details of patients’ demographics, medical history and medical usage were taken. In the beginning body height and weight, waist and hip circumferences were measured. Body mass index was calculated as body weight in kilograms divided by height in meters squared. Waist to hip ratio (WHR) was calculated as waist (cm)/hip (cm).

The propolis group received Iranian propolis capsules (500 mg twice daily, 30 kcal/day), whereas the placebo group received capsules with the same shape, color, and container. Placebo group also followed the same protocol as propolis group (twice daily, 30 kcal/day). It should be noted that placebo capsules were produced by the same company that manufactured the propolis capsules, containing all the ingredients except the active ingredient of propolis. In each group oral medication was administrated every 12 hours before meals with a glass of water for a period of 90 days. The diabetic diet and exercise regimen as baseline was continued without any change during the intervention period. At the beginning and the end of the study, Fasting and 2-hour post prandial (2hpp) blood samples were collected from the antecubital vein in the morning and the change in the biochemical parameters and inflammatory cytokines were evaluated.

*Dietary survey*

A 3-day dietary survey was taken using a 24 h recall method by experienced interviewers during the last week of study for the purpose of comparing dietary intake of energy and nutrients between two groups. The data were calculated based on Iranian Food Composition which was compiled by the Department of Nutrition, Tehran University of Medical Sciences, Tehran, Iran.

*Measurement of biochemical parameters*

Biochemistry analysis was carried out before and after the intervention in the Diabetes Research Center, Ahvaz Jundishapur University of Medical Sciences, Ahvaz, Iran. Concentration of serum glucose, creatinine (Cr), blood urea nitrogen (BUN), triglyceride (TG), Total cholesterol, high density lipoprotein-Cholesterol (HDL-C), low density lipoprotein-Cholesterol (LDL-C), very low-density lipoprotein-Cholesterol (VLDL-C) and uric acid were determined using commercial kit (Parsazmoon, Tehran, Iran) and Hitachi multianalyser (Hitachinaka, Japan) according to the manufacturer’s instructions. Also, the glycosylated hemoglobin (HbA1c) was measured using the Nycocard commercial HbA1c kit (oslo, Norway). Fasting insulin concentration was obtained by the use of enzyme-linked immunosorbent assay (Elisa) commercial kit (Monobind,California,USA). Fasting blood sugar (FBS) and insulin levels were used to calculate homeostasis model assessment-insulin resistance (HOMA-IR) and homeostasis model assessment of β-cell function (HOMA-β) as it comes below: HOMA-IR= glucose (mg/dl)*fasting insulin (µU/ml)/405 & HOMA-β=20* fasting insulin(µU/ml)/(Fasting glucose(mg/dl)-3.5).

eGFR was calculated using the Cockcroft-Gault Equation: eGFR= ((140-Age)/ (serum creatinine))*(weight/72) for males and for females the eGFR was multiplied by a correction factor of 0.85. Serum IL-1β, IL6 and TNF-α were measured by commercial Elisa kits (Diaclone, Besancon, France). High sensitive C-reactive protein (hs-CRP) also measured by a commercial Elisa kit (ZellBio, Ulm, Germany).

*Statistical Analysis*

Statistical analysis was performed using the SPSS 16 software (SPSS Inc., Chicago, IL, USA). The mean values are expressed as means ± standard deviation and checked for normality using Kolmogorov-Smirnov test before doing further analysis. When the data are in normality, Student’s t-test was used to analyze the difference between the two groups. Otherwise, Mann-Whitney rank sum test was accomplished. The Fisher’s exact test was used to evaluate the differences in categorical variables between the two groups. p < 0.05 was considered to indicate a statistically significant difference.


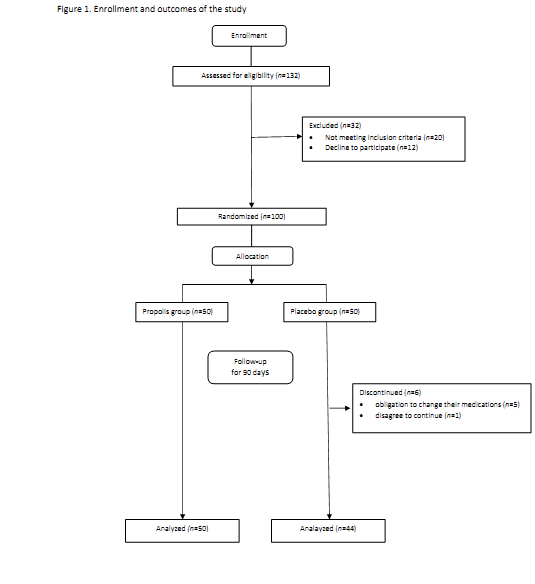


**Safety Considerations**

The candidates were informed of the study goals and procedures using the leaflet and signed written informed consent. Our research was performed in accordance with the Declaration of Helsinki. Supplements were licensed by the Iranian Ministry of Health and Medical Education. The protocol was approved by the Ethics Committee of Ahvaz Jundishapur University of Medical Sciences, Ahvaz, Iran, IR.AJUMS.REC.1396.430, and archived on the website of Iranian Ministry of Health ([www.irct.ir](http://www.irct.ir)) with the proprietary code of IRCT2016092730008N1.

The only adverse effect was reported by propolis is allergic reaction so the patients with any kind of allergies was excluded. Also severe renal dysfunction [estimated glumerular filtration rate (eGFR) <30 ml/min/1.73m2]; severe hepatic dysfunction (aspartate aminotransferase (AST) >100 U/I) or alanine aminotransferase (ALT) >100 U/I); serious cardiovascular and hematological disease and women in pregnancy or lactation were excluded.

**Follow-Up**

The propolis group received Iranian propolis capsules (500 mg twice daily, 30 kcal/day), whereas the placebo group received capsules with the same shape, color, and container. Placebo group also followed the same protocol as propolis group (twice daily, 30 kcal/day). It should be noted that placebo capsules were produced by the same company that manufactured the propolis capsules, containing all the ingredients except the active ingredient of propolis. In each group oral medication was administrated every 12 hours before meals with a glass of water for a period of 90 days. The diabetic diet and exercise regimen as baseline was continued without any change during the intervention period. At the beginning and the end of the study, Fasting and 2-hour post prandial (2hpp) blood samples were collected from the antecubital vein in the morning and the change in the biochemical parameters and inflammatory cytokines were evaluated.

**Data Management and Statistical Analysis**

Statistical analysis was performed using the SPSS 16 software (SPSS Inc., Chicago, IL, USA). The mean values are expressed as means ± standard deviation and checked for normality using Kolmogorov-Smirnov test before doing further analysis. When the data are in normality, Student’s t-test was used to analyze the difference between the two groups. Otherwise, Mann-Whitney rank sum test was accomplished. The Fisher’s exact test was used to evaluate the differences in categorical variables between the two groups. p < 0.05 was considered to indicate a statistically significant difference.

**Quality Assurance**

Patients with T2DM were screened and enrolled from the Department of Endocrinology, Golestan hospital, Ahvaz, Iran. T2DM was diagnosed in accordance with the criteria of the American Diabetes Mellitus Associations by an endocrinologist. The details of patients’ demographics, medical history and medical usage were taken by investigator.

For chemical proﬁle of propolis capsul which is characterized by the three parameters: total ﬂavones/ﬂavonols, ﬂavanones/dihydroﬂavonols, and phenolic compounds content that are used as a measure for the amount of active principles. According to investigation and based on statistics these amounts should be as following; ﬂavones/ﬂavonols: 8 ± 4%, ﬂavanones/dihydroﬂavonols: 6 ± 2%, total phenolic compounds: 28 ± 9% (20). Our samples were in the range (total flavones and flavonols: 8.4%, total flavanones and dihydroflavonols: 4.6% and total phenolic compounds: 28%).

**Expected Outcomes of the Study**

The broad spectrum of biological activity beside the long history of use and safety profile make propolis to be considered as a potential clinically useful drug **as** the study demonstrated that Iranian propolis has beneficial effects on reducing fasting blood glucose, post prandial blood glucose, serum insulin, insulin resistance and inflammatory cytokines also it can prevent the liver and renal dysfunction as well as can modulate lipid profile in T2DM patients.

**Dissemination of Results and Publication Policy**

Dr Narjes Zaeemzadeh will take the lead in publication and Dr Maryam Jenabi will take the responsibility to dissemination of results to participants.

**Duration of the Project**

| **20** | **19** | **18** | **17** | **16** | **15** | **14** | **13** | **12** | **11** | **10** | **9** | **8** | **7** | **6** | **5** | **4** | **3** | **2** | **Month 1** | **activity** |
| --- | --- | --- | --- | --- | --- | --- | --- | --- | --- | --- | --- | --- | --- | --- | --- | --- | --- | --- | --- | --- |
|  |  |  |  |  |  |  |  |  |  |  |  |  |  |  |  |  | ***** | ***** | ***** | Preprattion of propolis capsul and commercial kits |
|  |  |  |  |  |  |  |  |  |  |  |  |  |  | * | * | * |  |  |  | Study design and take the ethics cods |
|  |  |  |  |  |  |  | * | * | * | * | * | * | * |  |  |  |  |  |  | Collecting study subjects, blood samples, Dietary survey, etc |
|  |  | * | * | * | * |  |  |  |  |  |  |  |  |  |  |  |  |  |  | Measurement of biochemical parameters |
| * | * |  |  |  |  |  |  |  |  |  |  |  |  |  |  |  |  |  |  | Statistical analysis |

**Problems Anticipated**

The commercial kits were from Germany, USA and France and hardly available in Iran after the political issue the country was been through. Also our currency suddenly falling down and our budget was not enough anymore and we had to use personal budget to complete the study properly.

**Project Management**

Mehrnoosh Zakerkish: Endocrinologist and investigator that conducting the study.

Maryam Jenabi: design the study, interview and collect subjects, take Dietary survey, statistical analysis and write the article

Narjes Zaeemzadeh: corresponding for article, Take the responsibility of the budget and certificate of the project, and edit the article

Ali Asghar Hemmati: plan the study; initiate the whole idea of clinical trials

Niloofar Neisi: Measurement of biochemical parameters

**Ethics**

The candidates were informed of the study goals and procedures using the leaflet and signed written informed consent. Our research was performed in accordance with the Declaration of Helsinki. Supplements were licensed by the Iranian Ministry of Health and Medical Education. The protocol was approved by the Ethics Committee of Ahvaz Jundishapur University of Medical Sciences, Ahvaz, Iran, IR.AJUMS.REC.1396.430, and archived on the website of Iranian Ministry of Health ([www.irct.ir](http://www.irct.ir)) with the proprietary code of IRCT2016092730008N1.

**Informed Consent Forms**

The copy of informed consent form was attached to this file.

**Budget**

| **Made in** | **Company** | **Total Value**  **Rial** | **Value (each one)**  **Rial** | **number** | **Material** |
| --- | --- | --- | --- | --- | --- |
| Iran | Pars Azmoon | 000/500 | 000/250 | 2 | commercial kit FBS |
| Iran | Pars Azmoon | 000/500/1 | 000/500/1 | 1 | commercial kit TG |
| Iran | Pars Azmoon | 000/450 | 000/450 | 1 | commercial kit Chol |
| Iran | Pars Azmoon | 000/500/4 | 000/500/1 | 3 | Kit HDL-C |
| Iran | Pars Azmoon | 000/020/1 | 000/255 | 4 | Trucal U |
| Iran | Pars Azmoon | 000/400/2 | 000/600 | 4 | Trulab lipid |
| Iran | Pars Azmoon | 000/000/2 | 000/500 | 4 | Trucal HDL |
| Iran | Pars Azmoon | 000/400/1 | 000/350 | 4 | Trulab N |
| Iran | Pars Azmoon | 000/400/1 | 000/350 | 4 | Trulab P |
| Iran | Pars Azmoon | 000/700 | 000/700 | 1 | commercial kit ALT |
| Iran | Pars Azmoon | 000/700 | 000/700 | 1 | commercial kitAST |
| Iran | Pars Azmoon | 000/350 | 000/350 | 1 | commercial kit ALP |
| Iran | Pars Azmoon | 000/550 | 000/550 | 1 | commercial Urea kit |
| Iran | Pars Azmoon | 000/150 | 000/150 | 1 | commercial creatinin  kit |
| USA | Monobind | 000/000/15 | 000/000/5 | 3 | commercial kit Insulin |
| France | Diacolon | 000/000/30 | 000/000/15 | 2 | commercial kit IL-6 |
| France | Diacolon | 000/000/30 | 000/000/15 | 2 | commercial kitIL-1Β |
| France | Diacolon | 000/000/30 | 000/000/15 | 2 | commercial kit TNF-α |
| France | Diacolon | 000/000/16 | 000/000/8 | 2 | commercial kit hs-CRP |
| Iran | Shahdine Golha | 000/000/45 | 000/5 | 9000 | Propolos Capsul |
| Iran | Shahdine Golha | 000/001/8 | 900 | 9000 | Placebo Capsul |
| Iran |  | 000/300 | 000/150 | 2 box | Sampler |
| Iran |  | 000/450 | 000/150 | Box 3 | microtube |
| Iran |  | 000/000/2 | 5000 | 400 | Tube |
| Iran |  | 000/500 | 000/500 | Box 1 | Cup for BT3000 |
| Iran |  | 000/400 | 2000 | 200 | Vial CBC |
| Iran |  | 000/600 | 3000 | 200 | Syrin cc 5 |
| Iran |  | 000/600 | 3000 | 200 | Syring 2cc |
| Iran |  | 000/150 | 000/50 | Box 3 | Alcoholic Pad |

The total Budget that we evaluated about 1 years ago was around 200,000,000 Rials that was about 6500 dollars but as you know suddenly our currency fallen down and our budget was equal to 2000 dollars. So we had to spend the rest from personal budget to finish our study properly.


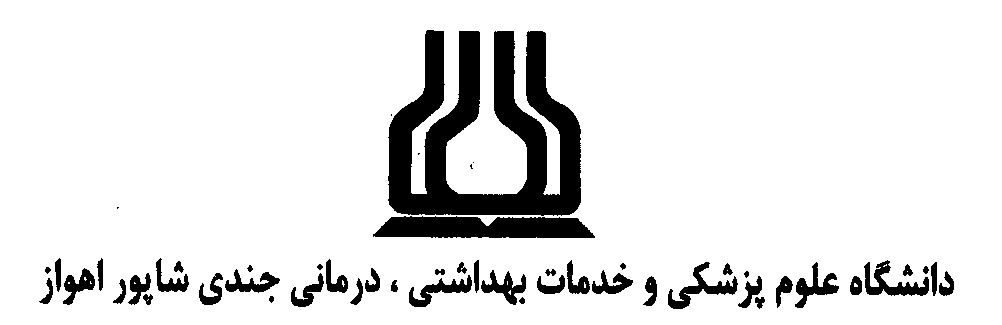


**Ahvaz Jundishapur Univercity of Medical Sciences**

**Diabetes Research Center**

**[Name of Principle Investigator]**

**Informed Consent form for Diabetic patients who attend Golestan Diabetes Clinic and who are inviting to participate in research on the effect of propolis in patients with type 2 diabetes mellitus.**

**Dr Mehrnoosh Zakerkish**

**Diabetes Research center**

**Ahvaz Jundishapur of Medical Sciences**

**The effect of propolis on glycemic control, Lipid profile, renal function and inflammatory biomarkers in patients with type 2 diabetes mellitus: A randomized double blind clinical Trial**

**This Informed Consent Form has two parts:**

- **Information Sheet (to share information about the research with you)**
- **Certificate of Consent (for signatures if you agree to take part)**

**You will be given a copy of the full Informed Consent Form**

**PART I: Information Sheet**

**Introduction**

I am Dr Mehrnoosh Zakerkish, Endocrinologist and Proffesor assistant in Medical school of Ahvaz Jundishapur University of Medical Sciences. We are doing research on Diabetes Mellitus, which is very common worldwide. I am going to give you information and invite you to be part of this research. You do not have to decide today whether or not you will participate in the research. Before you decide, you can talk to anyone you feel comfortable with about the research.

There may be some words that you do not understand. Please ask me to stop as we go through the information and I will take time to explain. If you have questions later, you can ask them of me, the study doctor or the staff.

**Purpose of the research**

Diabetes Mellitus (DM) is a common chronic disorder characterized by an elevated blood glucose concentration. The prevalence of diagnosed diabetes is increasing worldwide. According to the International Diabetes Federation the number of diabetic patients will be risen from 366 million in 2011 to 439 million by 2030 that cause mainly by the increasing western sedentary lifestyle and obesity. Poorly controlled diabetes is associated to complications which lead to significant decrease in life expectancy.

Propolis is a natural resinous hive product that honeybees (*Apis mellifera L*.) collect from various plants sources and mix with their salivary gland enzymes and wax. Propolis has a long history of being used in folk medicine dates back to ancient time in many countries and since then has been largely utilized as a medicine. many studies have shown it possesses antimicrobial, immunomudulatory, antitumor, anti-inflammatory, antioxidant, antiviral, antifungal as well as hepatoprotective, nephroprotective and pancreatoprotective activities. According to recent studies, propolis also has hypoglycemic activity and may have some positive effect on diabetic complications and improve the metabolism of blood lipid. So we decide to prove that Iranian Propolis has such good effect in diabetic patients.

**Type of Research Intervention**

The research will involve taking 90 days of Propolis capsule and having blood sample from you in the beginning and in the end of the research to see how much taking this supplement improve your health. And we are available in this period to see us any time. This research includes 3-month follow-up visits to clinic.

**Participant selection**

We are inviting all adult with type 2 diabetes mellitus that aged between 35-85 years, and receiving treatment with oral hypoglycemic agents.

.

- ***Example of question to elucidate understanding:*** *Do you know why we are asking you to take part in this study? Do you know what the study is about?*

**Voluntary Participation**

Your participation in this research is entirely voluntary. It is your choice whether to participate or not. Whether you choose to participate or not, all the services you receive at this clinic will continue and nothing will change. If you choose not to participate in this research project, you will offered the treatment that is routinely offered in this clinic for diabetes, and we will tell you more about it later. You may change your mind later and stop participating even if you agreed earlier*.*

- ***Examples of question to elucidate understanding:*** *If you decide not to take part in this research study, do you know what your options are? Do you know that you do not have to take part in this research study, if you do not wish to? Do you have any questions?*

**Information on the Trial Drug Propolis capsule**

1) give the phase of the trial and explain what that means. Explain to the participant why you are comparing or testing the drugs.

2) provide as much information as is appropriate and understandable about the drug such as its manufacturer or location of manufacture and the reason for its development.

3) explain the known experience with this drug

4) explain comprehensively all the known side-effects/toxicity of this drug, as well as the adverse effects of all the other medicines that are being used in the trial

The supplement we are testing in this research is called Propolis. It has been tested before with people and available in drug store. We now want to test this supplementation on people who have Type 2 Diabetes Mellitus.

The Propolis capsule is made by Shahdine Golha Company. You should know that it has a very few side effects. The main side effects, or problems, is that you may have allergic reaction.

Some participants in the research will not be given the drug which we are testing. Instead, they will be given the Placebo, the starch capsule which is most commonly used as food and has no effect and no side effect.

**Procedures and Protocol**.

**A. Unfamiliar Procedures**

Because we do not know if use of this supplementation is better for type 2 diabetes mellitus patients, we need to compare the two. To do this, we will put people taking part in this research into two groups. The groups are selected by chance, as if by tossing a coin.

Participants in one group will be given the Propolis capsule, while participants in the other group will be given the Placebo capsules. A placebo or inactive medicine looks like real medicine but it is not. It is a dummy or pretend medicine. It has no effect on a person because it has no real medicine in it. Sometimes when we want to know whether a new medicine is good, we give some people the new medicine and some people the pretend or dummy medicine. For the research to be good, it is important that you do not know whether you have been given the real medicine or the pretend or dummy medicine. This is one of the best ways we have for knowing what the medicine we are testing really does.

It is important that neither you nor we know which of the two capsules you are given. This information will be in our files, but we will not look at these files until after the research is finished. This is the best way we have for testing without being influenced by what we think or hope might happen. We will then compare which of the two has the best results.

The healthcare workers will be looking after you and the other participants very carefully during the study. If we are concerned about what Propolis is doing, we will find out which group you are getting and make changes. If there is anything you are concerned about or that is bothering you about the research please talk to me or one of the other researchers)

For any clinical study (if relevant):

We will take blood from your arm using a syringe and needle. Each time we will take about this much blood. In total, we will take about 5 cc, this much blood in the beginning and 3 month later in the end of the study. At the end of the research, in 1 year, any left-over blood sample will be destroyed

**B. Description of the Process**

During the research you make 5 visits to the clinic.

- In the first visit, a small amount of blood will be taken from your arm with a syringe. This blood will be tested for the presence of substances that show your healthy. We will also ask you a few questions about your general health and measure how tall you are and how much you weigh.
- At the next week, which will be 2 weeks later, you will be asked some questions about your health and then you will be given either the Proplis or placebo for one month. As explained before, neither you nor we will know whether you have received the test or the dummy/pretend drug.
- At the next visit, which will be 1 month later, you will again be asked some questions about your health and then you will be given either the Proplis or placebo for one month
- At the next visit, which will be 2 month later and 1 month after the previous visit, you will again be asked some questions about your health and then you will be given either the Proplis or placebo for one month.
- After 3 months from taking capsules, you will come back to the clinic for a blood test.

**Duration**

The research takes place over 104 days in total. During that time, it will be necessary for you to come to the clinic 5 days, for 3 hours each day. We would like to meet with you three months after your last clinic visit for a final check-up.

In total, you will be asked to come 6 times to the clinic in 6 months. At the end of six months, the research will be finished*.*

- ***Examples of question to elucidate understanding:*** *Can you tell me if you remember the number of times that we are asking you to come to the hospital to complete the treatment? The research projects? How many injections will you be given? How many tablets? How much blood will be taken from your veins, using a syringe and needle? Over how many weeks? Etc. Do you have any other questions? Do you want me to go through the procedures again?*

**Side Effects**

As already mentioned, this supplementation can have few unwanted effects. It can make you have allergic reaction. It is possible that it may also cause some problems that we are not aware of. However, we will follow you closely and keep track of any unwanted effects or any problems. We may use some other medicines to decrease the symptoms of the side effects or reactions. Or we may stop the use of one or more drugs. If this is necessary we will discuss it together with you and you will always be consulted before we move to the next step*.*

**Risks**

By participating in this research, it is possible that you will not be at any risk than you would otherwise be.

- ***Examples of question to elucidate understanding:*** *Do you understand that, while the research study is on-going, no-one may know which medicine you re receiving? Do you know that the medicine that we are testing is a new medicine, and we do not know everything about it? Do you understand that you may have some unwanted side-effects from the medicines? Do you understand that these side-effects can happen whether or not you are in the research study? Etc. Do you have any other questions?*

**Benefits**

If you participate in this research, you will have the following benefits: Antioxidant effect of propolis, reduce the blood glucose, reduce inflammation, and improve your health by making your immune system works stronger. There may not be any benefit to the society at this stage of the research, but future generations are likely to benefit*.*

**Reimbursements**

You will not be given any money or gifts to take part in this research.

- ***Examples of question to elucidate understanding:*** *Can you tell me if you have understood correctly the benefits that you will have if you take part in the study? Do you know if the study will pay for your travel costs and time lost, and do you know how much you will be re-imbursed? Do you have any other questions?*

**Confidentiality**

With this research, something out of the ordinary is being done in your community. It is possible that if others in the community are aware that you are participating, they may ask you questions. We will not be sharing the identity of those participating in the research.

The information that we collect from this research project will be kept confidential. Information about you that will be collected during the research will be put away and no-one but the researchers will be able to see it. Any information about you will have a number on it instead of your name. Only the researchers will know what your number is and we will lock that information up with a lock and key. It will not be shared with or given to anyone except my Ph.D. students Dr. Maryam Jenabi.

- ***Example of question to elucidate understanding:*** *Did you understand the procedures that we will be using to make sure that any information that we as researchers collect about you will remain confidential? Do you have any questions about them?*

**Sharing the Results**

The knowledge that we get from doing this research will be shared with you through community meetings before it is made widely available to the public. Confidential information will not be shared. There will be small meetings in the community and these will be announced. After these meetings, we will publish the results in order that other interested people may learn from our research.

**Right to Refuse or Withdraw**

You do not have to take part in this research if you do not wish to do so and refusing to participate will not affect your treatment at this clinic in any way. You will still have all the benefits that you would otherwise have at this clinic. You may stop participating in the research at any time that you wish without losing any of your rights as a patient here. Your treatment at this clinic will not be affected in any way.

**Alternatives to Participating**

If you do not wish to take part in the research, you will be provided with the same routine use of oral Diabetes drugs as you used before.

**Who to Contact**

If you have any questions you may ask them now or later, even after the study has started. If you wish to ask questions later, you may contact Dr. Maryam Jenabi; 09166344139; email: [jenabi_maryam@yahoo.com](mailto:jenabi_maryam@yahoo.com)

**This proposal has been reviewed and approved by The Ethics Committee of Ahvaz Jundishapur University of Medical Sciences, which is a committee whose task it is to make sure that research participants are protected from harm. Also, Ahvaz Jundishapur University of Medical Sciences is funding the study.**

- ***Example of question to elucidate understanding:*** *Do you know that you do not have to take part in this study if you do not wish to? You can say No if you wish to? Do you know that you can ask me questions later, if you wish to? Do you know that I have given the contact details of the person who can give you more information about the study? Etc.*

You can ask me any more questions about any part of the research study, if you wish to. Do you have any questions?

**PART II: Certificate of Consent**

**I have read the foregoing information, or it has been read to me. I have had the opportunity to ask questions about it and any questions that I have asked have been answered to my satisfaction. I consent voluntarily to participate as a participant in this research.**

**Print Name of Participant__________________**

**Signature of Participant ___________________**

**Date ___________________________**

**Day/month/year**

**If illiterate**

**I have witnessed the accurate reading of the consent form to the potential participant, and the individual has had the opportunity to ask questions. I confirm that the individual has given consent freely.**

**Print name of witness_____________________ AND Thumb print of participant**

**Signature of witness ______________________**

**Date ________________________**

**Day/month/year**

**Statement by the researcher/person taking consent**

**I have accurately read out the information sheet to the potential participant, and to the best of my ability made sure that the participant understands that the following will be done:**

**1.**

**2.**

**3.**

**I confirm that the participant was given an opportunity to ask questions about the study, and all the questions asked by the participant have been answered correctly and to the best of my ability. I confirm that the individual has not been coerced into giving consent, and the consent has been given freely and voluntarily.**

**A copy of this ICF has been provided to the participant.**

**Print Name of Researcher****/person taking the consent________________________**

**Signature of Researcher /person taking the consent__________________________**

**Date ___________________________**

**Day/month/year**

تاریخ: .................................................


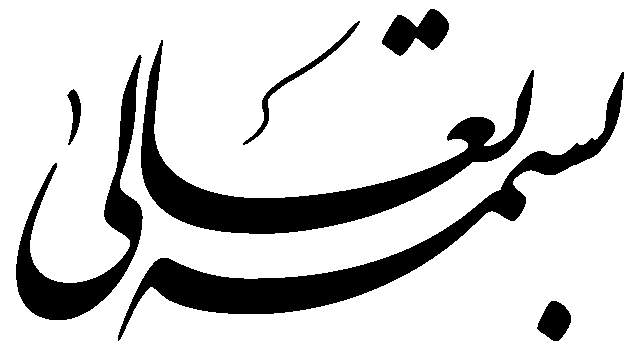

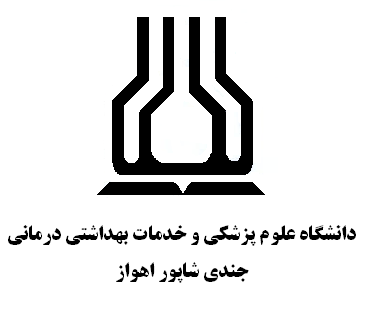


فرم رضایت آگاهانه

شرکت در طرح تحقیقاتی

| **عنوان طرح پژوهشي** | مطالعه تاثیر فراورده دارویی پروپولیس بر کنترل قند خون، پروفایل لیپیدی، شاخص های کلیوی و فاکتورهای التهابی سرم در بیماران دیابتی نوع 2: یک مطالعه کار­آزمایی بالینی تصادفی شده دو سو کور و محصول محور | |
| --- | --- | --- |
| **نام مجري** | **دکتر نرجس زعیم زاده** | |
| **معرفی پژوهش** | در این مطالعه به بیماران دیابتی نوع 2کپسول پروپولیس به مقدار mg500 دوبار در روز به مدت 90 روز داده خواهد شد تا تاثیر آن بروی قند خون ، پروفایل لیپید و عملکرد کلیه بررسی شود. | |
| **خونگیری** | نمونه خون قبل و بعد از مطالعه برای مقایسه تاثیر گذاری گرفته خواهد شد. | |
| **مزایا** | در صورت تاثیر گذاری پروپولیس بر قند خون با مطالعات بیشتر میتوان به عنوان یک داروی پایین آورنده قند خون به بیمار پیشنهاد شود.. | |
| **خطرات** | بروز حساسیت به پروپولیس | |
| **جبران خطرات** | درصورت بروز هرگونه عارضه احتمالی در هنگام مصرف جبران خسارت به عهده پژوهشگر خواهد بود | |
| **هزینه** | هزینه کلیه آزمایشات و دارو و دارونما به عهده پژوهشگر خواهد بود. | |
| **روشهای جایگزین** | میتواند دوزداروی پایین­آورنده را با نظر پزشک افزایش دهد. | |
| **محرمانه بودن** | اطلاعات شخصی کاملا محرمانه باقی مانده و نتایج مطالعه و آزمایشات در هر مرحله از مطالعه به اطلاع بیمار خواهد رسید. | |
| **پاسخگویی به پرسش‌ها** | آدرس و شماره تلفن پژوهشگر در اختیار بیماران قرار خواهد گرفت. | |
| **((رضايت))**  اينجانب با آگاهي کامل از موارد فوق و توضيحات حضوري مجري رضايت مي‌دهم که به عنوان يک فرد مورد مطالعه در پژوهش به صورت كاملا اختياري و آزاد شرکت نمايم.  کليه اطلاعاتي که از من گرفته مي‌شود و نيز نام من محرمانه باقي خواهد ماند و نتايج تحقيقات به صورت کلي و در قالب اطلاعات گروه مورد مطالعه منتشر مي‌گردد و نتايج فردي درصورت نياز بدون ذکر نام و مشخصات فردي عرضه خواهد گرديد و همچنين برائت پزشک يا پزشکان اين طرح را ازکليه اقدامات مذکور در برگه اطلاعاتي در صورت عدم تقصير درارائه اقدامات اعلام مي‌دارم.  اين موافقت مانع از اقدامات قانوني اينجانب در صورتي که عملي خلاف وغير انساني انجام شود نخواهد بود. | | |
| آدرس |  | |
| شماره تماس |  | |
| امضاء واثر انگشت فرد مورد پژوهش | | **امضاء پژوهشگر** |
| لطفا پيشنهادات و نظرات و يا مشكلاتي و هرگونه خلافي كه در پروسه انجام اين تحقيق وجودداشته يا دارد با شماره تلفن 3361984-0611 دفتر كميته اخلاق دانشگاه علوم پزشكي جندي شاپور اهواز و يا با پست الكترونيك [ethic@ajums.ac.ir](mailto:ethic@ajums.ac.ir) با ما در ميان بگذاريد. | | |

**Name**: Mehrnoosh

**Last name**: Zakerkish

**Date of Birth**: 1969/02/27

**Place of Birth**: Abadan

**Country**: Iran

**Present position**:

Assistant professor in faculty of medicine, Jundishapur Universityof Medical Sciences,Ahvaz_Iran, from 2009.

**Education**(s):

1. M.D. Ahvaz Jundishapur University of Medical Sciences, Ahvaz, Iran
2. Specialist in Internal Medicine, Ahvaz Jundishapur University of Medical Sciences, Ahvaz, Iran
3. Specialist in Endocrin disorder, Ahvaz Jundishapur University of Medical Sciences, Ahvaz, Iran

**Publications**

1. Blood pressure lowering and anti-inflammatory effect of hesperidin in type 2 diabetes

[Homayouni F](https://www.ncbi.nlm.nih.gov/pubmed/?term=Homayouni%20F%5BAuthor%5D&cauthor=true&cauthor_uid=29468764), [Haidari F](https://www.ncbi.nlm.nih.gov/pubmed/?term=Haidari%20F%5BAuthor%5D&cauthor=true&cauthor_uid=29468764), [Hedayati M](https://www.ncbi.nlm.nih.gov/pubmed/?term=Hedayati%20M%5BAuthor%5D&cauthor=true&cauthor_uid=29468764), [Zakerkish M](https://www.ncbi.nlm.nih.gov/pubmed/?term=Zakerkish%20M%5BAuthor%5D&cauthor=true&cauthor_uid=29468764), [Ahmadi K](https://www.ncbi.nlm.nih.gov/pubmed/?term=Ahmadi%20K%5BAuthor%5D&cauthor=true&cauthor_uid=29468764), [Phytother Res.](https://www.ncbi.nlm.nih.gov/pubmed/29468764) 2018 Feb 22

2. Association of Microalbuminuria and Estimated Glomerular Filtration Rate With Carotid Intima-Media Thickness in patients With Type 2Diabetes Mellitus, Hamid Dehdashti shahrokh,Mehrnoosh zaerkish,Aaraf Jenabi,Mohammad Ghasem Hanafi,Fakher Rahim,Afshin Rezazadeh,Heshmat allahshahbazian,FarzanehForouzan, Jentashapir J Health Res.2015April;6(2)

3. The Impact of Resveratrol Supplementation onBlood Glucose,Insulin,Insulin Resistance,Triglyceride,and Periodontal Markers in Type 2 Diabetic Patients with Chronic Periodontitis, Ahmad Zare Javid, Razie Hormoznejad, Hojat allah Yousefimanesh, Mehrnoosh Zakerkish, Mohammad Hosein Haghighi-zadeh, Parvin Dehghan and Maryam Ravanbakhsh, Phytotherapy research,Vol 31,Issue:1

4- Correlation between Non-Alcoholic Fatty Liver Disease and Carotid Intima-Media Thickness in patient with Type II Diabete, Mohammad Ghasem Hanafi,Masoud Cina,Mehrnoush Zakerkish,Fakher Rahim,Amal Saki-Malehi and Qasem Nissi, International Journal of Osteoporosis and Metabolic Disorders8(2):35-41.2015

5- Effect of Sesamin on the Glycemic Index, Lipid profile,and Serum Malondialde Level of Patients with Type II Diabetes, M. Mohammadshahi , M. Zakerzadeh , M. Zakerkish , M. Zarei, A. Saki, J Babol Univ Med Sci,Vol 18,Issu 6; Jun 2016

6- The Relationship between Serum Level of Vitamin D3 and the Severity of New Onset Rheumatoid Arthritis Activity, : Elham Rajaee , Ali Ghorbani , Karim Mowla , Mehrnoosh Zakerkish, Maryam Mohebi, Mehrdad Dargahi-MalAmir, journal of clinical and Diagnostic Research.2017 Mar,Vol-11(3):OC 28-OC30

7- Acute Aerobic Exercise and plasma Levels of Orexin A,Insulin,Glucose,and Insulin Resistance in Males with Type 2 Diabetes, Ali Akbar Alizadeh, Farhad Rahmani-Nia, Hamid Mohebbi, and Mehrnoosh Zakerkish, jundishapur j healthSci.2016 Janyary;8(1)

8- Evaluation ofl-carnitine Efficacy in the Treatment of Non-Alcoholic Fatty Liver Disease among diabetic patients:ARandomized Double Blind pilot Study, Pezhman Alavinejad, Mehrnoosh Zakerkish, Eskandar Hajiani, Seyed Jalal Hashemi, Mahmoud Chobineh, Elham Karimi Moghaddam, Journal of Gastroenterology and Hepatology Research2016 Ocotber;5(5)

9- Impact of cranberry juice enriched with omega-3 fatty acids adjunct with non-surgical periodontal treatment on metabolic control and periodontal status in type-2 diabetic patients with periodontal disease, Ahmad Zare Javid, Leila Maghsoumi-Norouzabad, Elnaz Ashrafzadeh, Hojat Allah Yousefimanesh, Mehrnoosh Zakerkish, Kambiz Ahmadi Angali, Maryam Ravanbakhsh & Hosein Babaei, Journal of the American College of Nutrition, 37(1):1-9

10- Etiology of Hirsutism in Women Referring to Endocrinology Clinic in Ahwaz, Hajieh Shahbazian,Mehrnoosh Zakerkish,Neda Heidari-Manesh, Zahedan journal of Research in Medical Science2013 Apr;15(4):69-72

11- Effect of Eight Weeks Aerobic Exercise on Plasma levels of Orexin A,Leptin,Glucose, Insulin , and Insulin Resistance in Maleswith Type 2 Diabetes, Ali Akbar Alizadeh, Farhad Rahmani-Nia , Hamid Mohebbi , Mehrnoosh Zakerkish, Iranian journal of diabetes and obesity, Vl 6, number 4,winter 2015

12- Albuminuria and Its Correlatesin type 2 diabetic patients, Mehrnoosh Zakerkish, Hajieh Bibi Shahbazian, Heshmatollah Shahbazian, Seyed Mahmoud Latifi, Armaghan Moravej Aleali, Iranian journal of Kidney disease, Vol 7, number4, july 2013

13- Association between Serum Vitamin D Level and Glycemic and Inflammatory Markers in Non-obese Patients with Type 2 Diabetes, Fatemeh Haidari, Mehrnoosh, Majid Karandish, Azadeh Saki,,Sakineh Pooraziz1, IJMS Vol 41, No 5, September 2016

14- : Effects of Concentrated Pomegranate Juice on Subclinical Inflammation and Cardiometabolic Risk Factors for Type 2 Diabetes: A QuasiExperimental Study, Farideh Shishehbor, Majid Mohammad Shahi, Mehdi Zarei,Azadeh Saki, Mehrnoosh Zakerkish,,Fatemeh Shirani, and Maryam Zare, Int J Endocrinol Metab. 2016 January; 14(1): e33835

15- Effect of various drug treatments on bone density in hypogonadal men, Hajieh Bibi Shahbazian, Karim Molaw, Asghar Zarea, Mehrnoosh Zakerkish,, Armaghan Moravej Aleal*i,* Oak j Med Sci 2012 Vol .28 No.4

16- Effect of sesamin supplementation on glycemic status,inflammatory markers,and adiponectin levels in patients with type 2 diabetes mellitus, Majid Mohammad shahi,Mehrnoosh zakerzadeh,Mehrnoosh zakerkish ,Mehdi Zarei,Azadeh Saki, journal of dietary supplements,00(00):1-11,2016

17- Serum level of vitamin D in new cases of Multiple Sclerosis, Shahram Rafie, Mehrnoosh Zakerkish,Seyed Aidin sajedi,seyedeh zeinab Razavi, vol7,issue-4,2016,pp 1623-1626

18- Effect of Ginseng extract on chemerin,Apelin and glycemic biomarkers in type 2 diabetic patients, Seyed Ahmad Hosseini, Ehsan Ghaedi, Mehrnoosh Zakerkish, Ata Ghadiri, Damoon Ashtary-larky, Maryam Safari, Mehdi Parsanahad and Meysam Alipour, Indian J physiol pharmacol 2017;61(2)

*19-* Hesperidin Supplementation Alleviates Oxidative DNA Damage and Lipid peroxidation in Type 2 Diabetes: A Randomized Double-Blind Placebo_Controlled Clinical Trial, Fatemeh Homayouni, Fatemeh Haidari, Mehdi Hedayati, Mehrnoosh Zakerkish, Kambiz Ahmad, PHYTOTHERAPY RESEARCH,31(10)

20- Comparative Efficacy of Duloxetine Versus Nortriptyline in Patients with Diabetic Peripheral Neuropathic pain:A Double Blind Randomized Controlled Trial, Mehrnoosh zakerkish,Fatemeh Amiri,Nastaran Majdi Nasab, Ali Ghorbani, Iran Red Crescent Med J.2017 August;19(8):e59995

21- The relationship between metabolic factors and quality of life aspects in type 2 diabetes patients, Haidari F,Mansoori E,Zakerkish M,Haghighizadeh, Vol 10, Issue 5, 2017

22- A Rare Constellation of Hurthle Cell Thyroid Carcinoma and parathyroid Carcinoma, Mehrnoosh zakerkish, Elham Rajaei, Mehrdad Dargahi, Mohammad Bahadoram, Journal of clinical and diagnostic research.2015 Dec,Vol-9(12): OD08-OD10

23- Astaxanthin improves glucose metabolism and reduces blood pressure in patients with type 2 diabetes mellitus, Mashhadi, Nafiseh Sokri ; Zakerkish, Mehrnoosh ; Mohammadiasl, Javad; Zarei, Mehdi; Mohammadshahi, Majid; Haghighizadeh, Mohammad Hossein, Asia Pacific journal of clinical nutrition ,VOL,NO:Vol 27,Issue 2,2018

24- The Effects of Active Ingredients of Barberry Root (Berberine) on Glycemic Control and Insulin Resistance in Type 2 Diabetic Patients, Rashidi, Homeira; Namjoyan, Foroogh; Mehraban, Zahra; Zakerkish, Mehrnoosh; Ghaderian, Seyed Bahman; Latifi, Seyed Mahmoud, JUNDISHAPUR JOURNAL OF NATURAL PHARMACEUTICAL PRODUCTS.2018 Feb,Vol 13(1)

25- Comparison different oral glucose lowering drugs effects on fasting patients with type II diabetes, Mehrnoosh Zakerkish1*, Hajiyeh Bibi Shahbazian1, Majid Karandish2, Homeira Rashidi1, Seyed Peyman Payami, International Journal of BioMedicine and Public Health, VOL.1,NO.2, ,2018: 59-66

**Name**: Maryam

**Last name**: Jenabi

**Date of Birth**: 1986/3/13

**Place of Birth**: Tehran

**Country**: Iran

**Education**(s):

1. Pharm. D.: Pharmacist, The School of Pharmacy, Tehran University of Medical Sciences
2. Ph.D.: Clinical Pharmacology, The School of Pharmacy, Ahwaz Jundishapur University of Medical Sciences

**Publications**

1- Serum tocopherol, retinol and ascorbic acid status in the healthy local population of Tehran,

Afshin Rakhsha, Sima Sadrai, Maryam Jenabi, Zahra Kazemi Ashtiani, Farnaz Barati, Mahboubeh Shaneshin, Mannan Hajimahmoodi; International Journal of Pharmacy & Technology; 2013; 5(1) 5289-5302.

2- Protective effect of saffron extract on vancomycin-induced nephrotoxicity, Maryam Jenabi, Ali Asghar Hemmati, Katayoon Hafezi, Esrafil Mansouri; Journal of nephrophropatology, accepted in May 2018.

**Presentations in domestic and international congresses:**

The effect of saffron extract on vancomycin-induced nephrotoxicity, International congress of physiology and pharmacology, 2018, Chabahar, Iran

**Teaching skills**:

Teaching pharmacology courses to medical, nursing, midwifery, nutrition, laboratory sciences, and Ph.D. students of chemistry, and nutrition in several faculties of Jundishapur University of Medical Sciences, Ahvaz,Iran

**Name**: Narjes

**Last name**: Zaeemzadeh

**Date of Birth**: 1975/9/7

**Place of Birth**: Mashhad

**Country**: Iran

**Present position**:

Assistant professor of Pharmacology in the faculty of medicine, Jundishapur Universityof Medical Sciences,Ahvaz_Iran, from 2009.

Education(s):

1. M.D; Rafsanjan Medical Sciences University Rafsanjan_Iran
2. Ph.D. of pharmacology; Ahwaz jundishapur University of Medical Sciences, Ahwaz, Iran

**Publication**

1. Protective Effect of Caffeic acid Phenethyl Ester (CAPE) on Amiodarone –Induced Pulmonary Fibrosis in Rat.

2- Effect of Aqueous Extract of Ruta graveolens on Spermatogenesis of Adult Rats.

3- Comparing the efficacy of four different protocols for eradicating of helicobacter pylori infection in Ahvaz, southwest of Iran.

4- Pharmacological knowledge loss among medical students in Ahvaz Jundishapur University of Medical Sciences

**Presentations in domestic and international congresses:**

1- Effect of curcumin & Lawson on kaolin-induced paw edema in rat. New Delhi, India, 1999.

2- Effect of clozapin on memory retention & retrieval in mouse. Shiraz,Iran, 2007.

3- Impact of Caffeic acid Phenethyl Ester (CAPE) on Amiodarone –Induced Pulmonary Fibrosis in Rat. Dresden, Germany, 2009.

4-A survey of zearalenone contamination of wheat in Ahwaz (west south of Iran). Sari, Iran, 2013

5- Effect of Aqueous Extract of Ruta graveolens on Spermatogenesis inAdult Male Rats. Ahvaz, Iran, 2014

6- Pharmacological knowledge loss in medical students of Jundi Shapour University of Medical Sciences. Kashan, Iran, 2015

7-Evaluation of the effect of Epigallocatechin gallate (green tea) on the serum levels of hepatic transaminases among patients with non-alcoholic fatty liver diseases: a randomized single blind clinical trial. Rome, Italy, 2017

**Name**: Ali Asghar

**Last name**: Hemmati

**Date of Birth**:1960/3/23

**Place of Birth**: Azna

**Country**: Iran

**Present position**:

Full Professor in Pharmacology, school of Pharmacy, Jundishapur Universityof Medical Sciences,Ahvaz_Iran, from 2009.

**Education**(s):

1. Pharm.D. Ahvaz Jundishapur University of Medical Sciences, Ahvaz, Iran
2. PhD in Pharmacology,The School of Pharmacy University of Bradford Richmond Road Bradford BD7 I DP England

**Publications**

1. [A study of the mechanisms underlying the anti-inflammatory effect of ellagic acid in carrageenan-induced paw edema in rats](javascript:void(0)), MT Mansouri, AA Hemmati, B Naghizadeh, SA Mard, A Rezaie, Indian journal of pharmacology, 2015, 47 (3), 292
2. [Protective effect of gallic acid against bleomycin-induced pulmonary fibrosis in rats](javascript:void(0)), J Nikbakht, AA Hemmati, A Arzi, MT Mansouri, A Rezaie, M Ghafourian, Pharmacological Reports, 2015, 67 (6), 1061-1067
3. [The preventive effect of atorvastatin on paraquat-induced pulmonary fibrosis in the rats](javascript:void(0)), MJ Khodayar, M Kiani, AA Hemmati, A Rezaie, MR Zerafatfard, Advanced pharmaceutical bulletin, 2014, 4 (4), 345
4. [Preventive effects of pomegranate seed extract on bleomycin-induced pulmonary fibrosis in rat](javascript:void(0)), AA Hemmati, A Rezaie, P Darabpour, Jundishapur journal of natural pharmaceutical products, 2013, 8 (2), 76
5. [Involvement of L-arginine/NO/cGMP/KATP channel pathway in the peripheral antinociceptive actions of ellagic acid in the rat formalin test](javascript:void(0)), B Ghorbanzadeh, MT Mansouri, AA Hemmati, B Naghizadeh, SA Mard, Pharmacology Biochemistry and Behavior, 2014, 126, 116-121
6. [Wound healing properties of quince seed mucilage: in vivo evaluation in rabbit full-thickness wound model](javascript:void(0)), P Tamri, A Hemmati, MG Boroujerdnia, International Journal of Surgery, 2014, 12 (8), 843-847
7. [Evaluation of the effects of caffeic acid phenethyl ester on prostaglandin E2 and two key cytokines involved in bleomycin-induced pulmonary fibrosis](javascript:void(0)), A Larki-Harchegani, AA Hemmati, A Arzi, M Ghafurian-Boroojerdnia, Iranian journal of basic medical sciences, 2013, 16 (7), 850
8. [Topical grape (Vitis vinifera) seed extract promotes repair of full thickness wound in rabbit](javascript:void(0)), AA Hemmati, N Aghel, I Rashidi, A Gholampur‐Aghdami, International wound journal, 2011, 8 (5), 514-520
9. [Involvement of L-arginine/NO/cGMP/KATP channel pathway in the peripheral antinociceptive actions of ellagic acid in the rat formalin test](javascript:void(0)), B Ghorbanzadeh, MT Mansouri, AA Hemmati, B Naghizadeh, SA Mard, Pharmacology Biochemistry and Behavior, 2014, 126, 116-121
10. [Wound healing properties of quince seed mucilage: in vivo evaluation in rabbit full-thickness wound model](javascript:void(0)), P Tamri, A Hemmati, MG Boroujerdnia, International Journal of Surgery, 2014, 12 (8), 843-847
11. [Protective effect of caffeic acid phenethyl ester (CAPE) on amiodarone-induced pulmonary fibrosisin rat](javascript:void(0)), N Zaeemzadeh, A Hemmati, A Arzi, M Jalali, I Rashidi, Iranian journal of pharmaceutical research: IJPR, 2011, 10 (2), 321
12. [The γ-secretase blocker DAPT impairs recovery from lipopolysaccharide-induced inflammation in rat brain](javascript:void(0)), S Nasoohi, AA Hemmati, F Moradi, A Ahmadiani, Neuroscience, 2012, 210, 99-109
13. [Healing effect of quince seed mucilage on T-2 toxin-induced dermal toxicity in rabbit](javascript:void(0)), AA Hemmati, H Kalantari, A Jalali, S Rezai, HH Zadeh, Experimental and toxicologic pathology,2012, 64 (3), 181-186
14. Hemmati A.A., Zaeemzadeh N. , Arzi A., Jalali T., Rashidi I. Evaluation of the effect of cafeic acid phenethyl ester (CAPE) on amiodarone-induced pulmonary fibrosis in rat. Iranain J Pharmaceutical Res 2009; (in press)
15. Hemmati A.A., Mikailil P., Khodayar M.J., Ghafurian M., Rashidi I. The protective effect of cetirizine against bleomycin induced pulmonary fibrosis in rat. Pakistan J Med Sci 2008 ; 24: 813- 820
16. Hemmati A.A., Nazari z, Ranjbari N, Torfi A. Comparison of the preventive effect of vitamin C and E on hexavalent chromium-induced pulmonary fibrosis in rat. Inflammopharmacology 2008; 16: 195-197 4
17. Hemmati A A., Nazari Z, Samei M. A comparative study of grape seed extract and vitamin E effects on silica-induced pulmonary fibrosis in rats. Pulmonary Pharmacology & Therapeutics 2008; 21: 668-674
18. Hemmati A.A., Rashidi I., Jafari M. Promotion of wound healing by Hypericum perforatum extract in rabbit. Jundishapur J Natural Pharmaceutical Products 2008; 2: 78-86
19. Hemmati AA, Nazari Z, Rashidi I, Kazemi Z. Protective effect of ketotifen on bleomycin-induced pulmonary fibrosis in rat. J Pharmacy Chemistry 2008; 2: 65-68.
20. Moazedi A.A., Belaran M., Hemmati A.A., Rasekh A. Co-Administration of epinephrine and glucose do not have synergic effects on the improvement of spatial learning task in young male rats. J Med Sci 2008; 8(1): 22-27
21. Moazedi A.A., Belaran M., Hemmati A.A., Rasekh A. The role of beta–adrenergic system on the enhancement of spatial learning caused by glucose injection in young male rats. Int J Pharmacol 2008; 4(1): 34-39
22. Fathi Moghaddam H, Hemmati AA, Nazari Z, Mehrab H, Abid KM, Shafiee Ardestani M. Effects of aspirin and celecoxib on rigidity in a rat model of parkinson`s disease. Pakistan J Biol Sci 2007; 10 (21): 3853-3858,
23. Saeidian SR, Hemmati AA, Haghighi MH. Pain relieving effect of short-course, pulse prednisolone in managing frozen shoulder. J Pain Palliat Care Pharmacother. 2007;21(1): 27-30.
24. Sharifipour F, Zamani M, Idani E, Hemmati AA. Oxygen therapy for severe corneal alkali burn in rabbits. Cornea 2007; 26: 1107-1110
25. Arzi A., Hemmati A.A., Ghobishavi S, Arzi L. The effect of L-tyrosine, vitamins E and C on perphenazine-induced catatonia in rat. Jundishapur J Natural Pharmaceutical Products 2007; 2: 18-24
26. Hemmati A.A., Aghel N., Nazari Z, Mohammadian B, Hasanvand N. Grape Seed Extract Can Reduce the Fibrogenic Effect of Bleomycin in Rat Lung. Iranian Journal of Pharmaceutical Sciences 2006; 2(3): 142-150
27. Arzi A., Hemmati A.A., Pipelzadeh M.H., Ramesh F., The effect of selegiline and bromocriptine in the prophylaxis of perphenazine-induced pseudoparkinsonim in rat: A comparative study. Jundishapur J Natural Pharmaceutical Products 2006; 1: 36-40
28. Moghbel A., Hemmati A.A., Agheli H., Rashidi I.1 , Amraee K. The effect of tragacanth mucilage on the healing of full thickness wound in Rabbit. Arch Iranian Med (2005) 8(4): 257-262
29. Pipelzadeh M.H., Hemmati A.A., Dezfulian A., Koochaek M.H., Rostami A. The role of Prostaglandins and mast cells in the modulation of acute acid-induced tracheal contraction in rat. Acta Medica Iranica (2004) 42: 31-35
30. Arzi A., Hemmati A.A., Razian A. Effect of vitamin C and E on cognitive function in mouse. Pharmacological research (2004) 49: 249-252 Hemmati A.A., Arzi A., Amin M. Effect of achillea millefolium extract in wound healing of rabbit 5 J. Natural Remedies 2002; 2: 164-167
31. Arzi A., Hemmati A.A., Amin M. Stimulation of wound healing by licorice in rabbit. Saudi Pharmaceutical Journal (2003) 11: 57-60 Hemmati A.A., Nazari Z., Motlagh M., Goldasteh S. The role of sodium cromolyn in treatment of paraquat-indued pulmonary fibrosis in rat. Pharmacological Research 2002; 46: 229-234
32. Hemmati A.A., Mahammadian F., An investigation into the effect of mucilage of quince seeds on wound healing in rabbit. Journal of Herbs, Spices and Medicinal Plants (2000) 7: 41-46.
33. Hemmati A.A., Hicks R., Increased myofibroblast contractile sensitivity in paraquat pretreated rat lung. Life Sciences (1999) 65: 2325-2332

Name: Niloofar

Last name: Neisi

Date of Birth: 1979

Place of Birth: Ahwaz

Country: Iran

Education(s):

1. B.Sc.: Laboratory Sciences, The School of Medicine, Ahwaz Jundishapur University of Medical Sciences
2. M.Sc.: Medical Virology, The School of Medicine, Ahwaz Jundishapur University of Medical Sciences
3. Ph.D.: Medical Virology, The School of Medicine, Ahwaz Jundishapur University of Medical Sciences

**Publications**

[The influence of substrate topography and biomaterial substance on skin wound healing.](https://www.ncbi.nlm.nih.gov/pubmed/26770875)

Ghanavati Z, **Neisi N**, Bayati V, Makvandi M. Anat Cell Biol. 2015 Dec;48(4):251-7. doi: 10.5115/acb.2015.48.4.251. Epub 2015 Dec 21. PMID: 26770875

[Study of the Association of Mutant HBsAg Gene and Hodgkin and Non-Hodgkin Lymphoma.](https://www.ncbi.nlm.nih.gov/pubmed/26862382)

Makvandi K, Ranjbari N, Makvandi M, Ashraf Teimori A, **Neisi N**, Rasti M, Alipour V, Albokord M, Kanani M, Ahadi R, Habibian A. Jundishapur J Microbiol. 2015 Nov 21;8(11):e25726. doi: 10.5812/jjm.25726. eCollection 2015 Nov. PMID:26862382

[Molecular Characterization of Pre-Core/Core and S Region of Hepatitis B Virus in Hemodialysis Patients With Occult Hepatitis B Infection.](https://www.ncbi.nlm.nih.gov/pubmed/26587212)

Rastegarvand N, Makvandi M, Samarbafzadeh A, Rasti M, **Neisi N**, Pouremamali A, Teimoori A, Shabani A. Jundishapur J Microbiol. 2015 Oct 26;8(10):e23686. doi: 10.5812/jjm.23686. eCollection 2015 Oct. PMID: 26587212

[Evaluation of hepatitis B surface antibody and specific gamma interferon response in health care workers after vaccination.](https://www.ncbi.nlm.nih.gov/pubmed/25789124)

Sarmast Shooshtari MH, Makvandi M, Rasti M, **Neisi N**, Rastegarvand N, Pouremamali A, Sadeghi Haj M, Ghaedi F. Jundishapur J Microbiol. 2014 Dec 6;8(1):e13801. doi: 10.5812/jjm.13801. eCollection 2015 Jan. PMID: 25789124

[Serotype determination of adenoviruses in children with respiratory infection.](https://www.ncbi.nlm.nih.gov/pubmed/24522944)

Ghasemi Y, Makvandi M, Samarbafzadeh AR, Nejati A, Najafifard S, **Neisi N**, Rasti M, Ahmadi K, Shamsizadeh A, Nikfar R. Indian J Pediatr. 2014 Jul;81(7):639-43. doi: 10.1007/s12098-013-1286-2. Epub 2014 Feb 14. PMID: 24522944

[Antisecretory effect of hydrogen sulfide on gastric acid secretion and the involvement of nitric oxide.](https://www.ncbi.nlm.nih.gov/pubmed/24707486) Mard SA, Askari H, **Neisi N**, Veisi A. Biomed Res Int. 2014;2014:480921. doi: 10.1155/2014/480921. Epub 2014 Feb 24. PMID: 24707486

[Occult hepatitis B virus among the patients with abnormal alanine transaminase.](https://www.ncbi.nlm.nih.gov/pubmed/25485052)

Makvandi M, **Neisi N**, Khalafkhany D, Makvandi K, Hajiani E, Shayesteh AA, Masjedi Zadeh A, Sina AH, Hamidifard M, Rasti M, Aryan E, Ahmadi K, Yad Yad MJ. Jundishapur J Microbiol. 2014 Aug;7(8):e11648. doi: 10.5812/jjm.11648. Epub 2014 Jul 13. PMID:25485052

[Detection of Hepatitis C virus RNA in peripheral blood mononuclear cells of patients with abnormal alanine transaminase in Ahvaz.](https://www.ncbi.nlm.nih.gov/pubmed/25008816) Makvandi M, Khalafkhany D, Rasti M, **Neisi N**, Omidvarinia A, Mirghaed AT, Masjedizadeh A, Shyesteh AA. Indian J Med Microbiol. 2014 Jul-Sep;32(3):251-5. doi: 10.4103/0255-0857.136553. PMID: 25008816

[MiR-328 May be Considered as an Oncogene in Human Invasive Breast Carcinoma.](https://www.ncbi.nlm.nih.gov/pubmed/28203454)

Saberi A, Danyaei A, **Neisi N**, Dastoorpoor M, Tahmasbi Birgani MJ.

Iran Red Crescent Med J. 2016 Nov 7;18(11):e42360. doi: 10.5812/ircmj.42360. eCollection 2016 Nov. PMID: 28203454

.

[Study on Rotavirus Infection and Its Genotyping in Children Below 5 Years in South West Iran.](https://www.ncbi.nlm.nih.gov/pubmed/27307959) Azaran A, Makvandi M, Samarbafzadeh A, **Neisi N**, Hoseinzadeh M, Rasti M, Teymurirad M, Teimoori A, Varnaseri M, Makvandi K. Iran J Pediatr. 2016 Mar 5;26(2):e2080. doi: 10.5812/ijp.2080. eCollection 2016 Apr.PMID:27307959

[Prevalence of Hepatitis C virus Genotype 3a in patients with Hodgkin and Non-Hodgkin Lymphoma.](https://www.ncbi.nlm.nih.gov/pubmed/28491250) Radmehr H, Makvandi M, Samarbafzadeh A, Teimoori A, **Neisi N**, Rasti M, Abasifar S, Soltani H, Abbasi S, Kiani H, Mehravaran H, Azaran A, Shahani T.

Iran J Microbiol. 2016 Dec;8(6):389-394. PMID: 28491250

[Protective activity of crocin against indomethacin-induced gastric lesions in rats.](https://www.ncbi.nlm.nih.gov/pubmed/26439477)

Mard SA, Pipelzadeh MH, Teimoori A, **Neisi N**, Mojahedin S, Khani MZ, Ahmadi I.

J Nat Med. 2016 Jan;70(1):62-74. doi: 10.1007/s11418-015-0938-0. Epub 2015 Oct 6.

PMID:   26439477

[Characterization of A Three-Dimensional Organotypic Co-Culture Skin Model for Epidermal Differentiation of Rat Adipose-Derived Stem Cells.](https://www.ncbi.nlm.nih.gov/pubmed/27602310) Ghanavati Z, Orazizadeh M, Bayati V, Abbaspour MR, Khorsandi L, Mansouri E, **Neisi N**. Cell J. 2016 Fall;18(3):289-301. Epub 2016 Aug 24. PMID:27602310

[Designing, Construction and Expression of a Recombinant Fusion Protein Comprising the Hepatitis E Virus ORF2 and Rotavirus NSP4 in the Baculovirus Expression System.](https://www.ncbi.nlm.nih.gov/pubmed/28138375)

Makvandi M, Teimoori A, **Neisi N**, Samarbafzadeh A. Jundishapur J Microbiol. 2016 Oct 8;9(11):e40303. doi: 10.5812/jjm.40303. eCollection 2016 Nov. PMID: 28138375

[The Prevalence of SEN Virus and Occult Hepatitis B (OBI) Virus Infection Among Blood Donors in Ahvaz City.](https://www.ncbi.nlm.nih.gov/pubmed/27679708) Abbasi S, Makvandi M, Karimi G, **Neisi N**. Jundishapur J Microbiol. 2016 Jul 3;9(7):e37329. doi: 10.5812/jjm.37329. eCollection 2016 Jul. PMID: 27679708

[Influence of L-carnitine on the Expression Level of Adipose Tissue miRNAs Related to Weight Changes in Obese Rats.](https://www.ncbi.nlm.nih.gov/pubmed/29023027) Nazari M, Saberi A, Karandish M, **Neisi N**, Jalali MT, Makvandi M. Pak J Biol Sci. 2016;19(5):227-232. doi: 10.3923/pjbs.2016.227.232.

PMID:29023027

[Three cases of mumps virus and enterovirus coinfection in children with enteroviral meningitis.](https://www.ncbi.nlm.nih.gov/pubmed/27930588) Rasti M, Makvandi M, **Neisi N**, Azaran A, Rastegarvand N, Khalafkhany D, Jahangirnezhad E, Teimoori A, Hadian M, Shabani A, Shamsizadeh A, Nikfar R, Varnaseri M. Medicine (Baltimore). 2016 Dec;95(49):e5610.PMID:27930588

[Molecular epidemiology of hepatitis A virus in patients in the Ahwaz region of Iran.](https://www.ncbi.nlm.nih.gov/pubmed/22337296)

Nejati A, Makvandi M, Samarbafzadeh A, **Neisi N**, Moradzadegan H. J Med Virol. 2012 Apr;84(4):582-6. doi: 10.1002/jmv.23238. PMID:22337296

[β-carotene protects the gastric mucosa against ischemia-reperfusion injury in rats.](https://www.ncbi.nlm.nih.gov/pubmed/23291590)

Seyyed Ali M, **Neisi N**, Darbor M, Hassanpour M, Makvandi M, Solgi G.

Eur Cytokine Netw. 2012 Oct-Dec;23(4):173-8. doi: 10.1684/ecn.2012.0317.

PMID: 23291590

[Gastroprotective effect of NaHS against mucosal lesions induced by ischemia-reperfusion injury in rat.](https://www.ncbi.nlm.nih.gov/pubmed/22271414)Mard SA, **Neisi N**, Solgi G, Hassanpour M, Darbor M, Maleki M.

Dig Dis Sci. 2012 Jun;57(6):1496-503. doi: 10.1007/s10620-012-2051-5. Epub 2012 Jan 22.
